# Unpacking media bias in the growing divide between cable and network news

**DOI:** 10.1038/s41598-025-01046-7

**Published:** 2025-05-21

**Authors:** Homa Hosseinmardi, Samuel Wolken, David M. Rothschild, Duncan J. Watts

**Affiliations:** 1 University of California Los Angeles, Los Angeles, CA 90024 USA; 2 University of Pennsylvania, Philadelphia, PA 19104 USA; 3 Microsoft Research New York, New York, NY 10012 USA

**Keywords:** Media effects, Polarization, Public opinion, Human behaviour, Computer science

## Abstract

The potential for a large, diverse population to coexist peacefully is thought to depend on the existence of a public sphere in which citizens are exposed to similar facts about similar topics. A generation ago, broadcast television news was widely considered to serve this function; however, since the rise of cable news in the 1990s, critics and scholars have worried that the corresponding fragmentation and segregation of audiences has caused this baseline of common understanding to be lost. Recent work documents that millions of Americans are loyal consumers of cable TV news stations. However, the implications of partisan segregation in TV news consumption depend on bias in content—which topics TV news programs talk about and the language they use to talk about them. Here, we measure bias in the production of TV news at scale by analyzing nearly a decade of TV news (Dec. 2012–Oct. 2022) on the largest cable and broadcast stations. We quantify the share of attention each station devoted to more than 20 politically significant topics as well as the linguistic similarity of different stations’ news coverage of those topics. We find that while broadcast news continues to cover similar topics with similar language, cable news stations have become increasingly distinct, both from broadcast news and from each other, diverging in terms of both content and language. This trend is driven by hard news as much as partisan commentary programs. Our results show that changes in the supply, not just consumption, of TV news are contributing to Americans’ polarizing media diets.

**Supplementary Information:**

The online version contains supplementary material available at 10.1038/s41598-025-01046-7.

## Introduction

As recently as the 1970s, an estimated 27-29 million people (out of a population of around 200 million) tuned in on any given weekday to watch Walter Cronkite deliver the news on CBS^1^. Along with his peers at ABC and NBC—which together comprised the “big three” of broadcast television—Cronkite’s dominance arguably created a common baseline of understanding for a substantial portion of the politically informed and active U.S. population. Even if they disagreed about the meaning of what they heard or what they should do about it, for 30 minutes every evening Americans across the political spectrum consumed the same set of facts about the same set of events, many of them from the same trusted individual. Scholars have argued that this common experience or “shared reality” had important consequences for social integration. By providing a baseline of shared knowledge and experience, it served as a sort of social, cultural, and political glue^2^—which, in turn, fostered among citizens a sense of civic engagement, shared national identity, and empathy^3^. Relatedly, scholars have also argued that citizens rely on the media for information and commentary on issues of the day, competing political perspectives, and insight into the conduct of public officials^4^. Thus, a shared media reality should also enhance support for bipartisanship^5^ and democratic norms among voters^6^.

In 2018, former President Barack Obama claimed that this shared media reality was gone, describing partisan media consumers as “living on another planet” and concluding that “one of the biggest challenges we have to our democracy is the degree to which we don’t share a common baseline of facts”^7^. Obama’s comment echoed broadly shared concerns among scholars of media and democracy that a growing number of Americans have homogeneous news diets focused on biased sources—in other words, they live in separate realities. As we will discuss in more detail below, however, many of these concerns have focused on the mechanisms by which a declining common baseline of understanding as a result of increasingly separate realities described on commonly consumed news might have caused outcomes such as increasing polarization or diminishing trust in institutions. Less attention has been paid to the descriptive question of whether or not separate realities described on commonly consumed news has indeed become more prevalent and, if so, to what extent and in what manner. Moreover, the bulk of descriptive analyses that have been performed have focused on online environments in reaction to earlier concerns about the fragmentation of the mass public into “echo chambers” or “filter bubbles”^8,9^. Interestingly, most of these analyses have found that Americans’ online news consumption is surprisingly low^10^, is generally diverse^11–17^, and that at most a tiny percentage of people plausibly “live” in echo chambers^18^. In other words, in spite of the huge increase in overall online media consumption over the past few decades, in the context of news specifically, online consumption itself is unlikely to have had much impact on the average Americans’ exposure to political information.

A more likely culprit for the loss or otherwise of a common factual baseline is television, which continues to account for roughly five times as much news consumption for average Americans than all online sources combined^10^. Recent work has found that roughly ten times as many Americans have partisan-segregated television news diets compared with online news consumption^18^, and that approximately 15% of Americans spend at least 8 hours per month watching partisan TV news with most partisan TV news consumers predominately watching a single channel^19^. However, fragmentation in TV news consumption does not necessarily signal the loss of a shared baseline of facts. It could be that the news that Americans consume from different sources is largely interchangeable in terms of selection and framing of topics. Or that “hard news” programs are relatively homogeneous across cable news stations, undercutting the polarizing effects of partisan commentary. Thus, the implications of Americans’ one-sided TV news diets depend on the content of news programs.

By some measures, the degree of bias in TV news has increased in recent years^20^, but to date no work has investigated the constituent elements of bias in TV news at scale. In particular, few descriptive empirical studies differentiate between bias arising from the selection of topics and bias arising from how these topics are covered, which are known to have different implications for viewers’ political opinions^21^.

In this paper, we offer a highly granular and comprehensive descriptive account of the U.S. television news ecosystem that (a) includes both broadcast and cable stations, allowing for within-broadcast, within-cable, and broadcast-cable comparisons; (b) separately accounts for topic selection and polarization of language. We examine the degree to which (a) stations exhibit selection bias, meaning that they allocate different levels of attention to potential topics, and (b) stations exhibit polarization in how they talk about the same topic, meaning that their discussion of a given topic features language that is different from other stations’ coverage of the same topic.

This paper complements recent work documenting the high degree of partisan segregation in TV news consumption^18,19^. The implications of “echo chambers” in TV news consumption depend on the degree of media bias present in newscasts, which we show has become pronounced in recent years. Cable TV news outlets are covering different topics and, when they do cover the same topics, using less similar language in how they talk about those topics. We show that CNN and MSNBC have become more similar in both topic selection and the language they use to talk about those topics, whereas FNC has diverged on both dimensions. And, recent increases in bias on cable TV news are due as much to hard news as they are to opinion news programs. We conclude with a brief discussion of the implications of our findings and possible directions for future work.

## Results

### Topic selection across stations

To estimate the presence and severity of bias in topic selection across different stations, we computed the proportion of news airtime devoted by each station to 24 topics that cover a combination of socially polarizing issues (e.g. abortion, immigration, gun, climate change), issues that became salient due to specific events (e.g. vaccination, China, Russia), and issues that are of perennial relevance to U.S.politics (e.g. healthcare, energy, education, the economy) and society (e.g. disasters, drugs, crime). See Supplementary Materials section B for more details and Table S7 for the list of topics. To obtain these estimates, we first coded all 13, 446, 736 news segments using a novel two-layered human-in-the-loop classification model that achieved an average precision of 78.45% (see Materials and Methods and Supplementary Materials for details). We then estimated the proportion of coverage that is about a certain topic, *k*, on a given day by dividing the total number of words of all segments labeled as topic *k* by the total number of words in that station’s news coverage on the same day (see Materials and Methods for more information about the classification process). Together, the selected 24 topics account for 55.1% of news segments from cable and 27.6% of news segments from broadcast networks, Fig. S3.

**Fig. 1:**
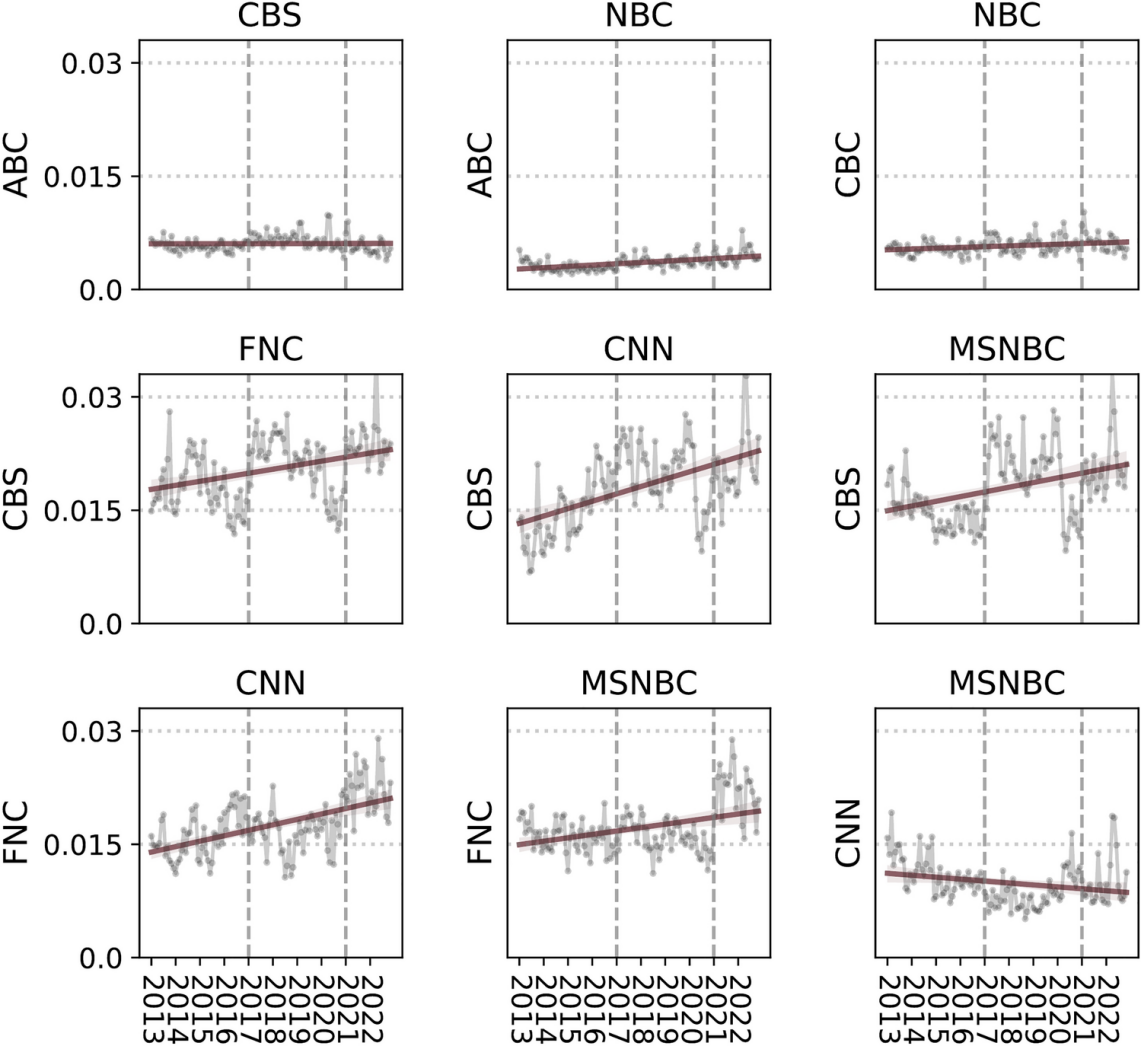
Average differences in coverage (fraction of time) for the 24 topics between all six stations.

Figure 1 shows the degree of aggregate variation in topic selection—i.e. between-station differences aggregated over all 24 topics—or whether such variation increased over time. For each pair of stations *i* and *j* and at each time *t*, we computed the aggregate difference in topic selection, $\delta ^{(i,j)} (t) = \sum _{k=1}^{K} |y^{i}_k(t)-y^{j}_k(t)| / K$, where $y^{i}_{k}($t) is the proportion of time devoted by station *i* to $k^{\text {th}}$ topic at time *t*, and $K=24$ is total number of topics. The top row of Fig. 1 shows differences in topic selection among the broadcast news programs: CBS vs. ABC (left); NBC vs. ABC (middle); and NBC vs. CBS (right). In all cases, the pairwise difference is small and not increasing over the time period.

 Next, the second row of Fig. 1 compares each of the three cable networks to CBS national news programming. (Here, we use CBS as the reference station, but given the similarity between the broadcast networks, results do not change when CBS is replaced by NBC or ABC.) In contrast with the between-broadcast comparisons in the top row, there are large differences in topic selection between broadcast news and all three cable networks—differences that are increasing over time. FNC is the most distinct overall, both starting and ending the ten-year period with higher values of $\delta ^{(i,j)}$ than either CNN or MSNBC. Perhaps surprisingly, both MSNBC and especially CNN show steeper increases over the interval: in 2012, MSNBC and CNN had more similar topic selection to CBS than FNC did; however, by 2022, all three cable networks are similarly distinct from broadcast news. Finally, the bottom row of Fig. 1 shows pairwise differences in topic selection between the cable networks: CNN vs. FNC (left); MSNBS vs. FNC (middle); and MSNBC vs. CNN (right). While the differences between the cable networks are not as large as the differences between cable and the broadcast networks, as expected, both CNN and MSNBC display substantial differences with FNC that have grown with time. In contrast, CNN and MSNBC were relatively similar in 2012 and have grown more similar since then.

To summarize, when comparing cable to broadcast news, we find that after the 2016 and 2020 U.S. presidential elections, cable networks’ topic coverage diverged sharply from broadcast news, reflecting a cyclical pattern tied to presidential campaigns and administrations. Among cable networks, FNC shows increasing divergence from CNN and MSNBC over time, particularly starting after 2020 and persisting throughout Biden’s presidency. In contrast, the comparison within broadcast networks shows only small fluctuations over the ten-year study period, as these stations maintain relatively small and stable divergence from one another in their selection of topics to cover.

**Fig. 2:**
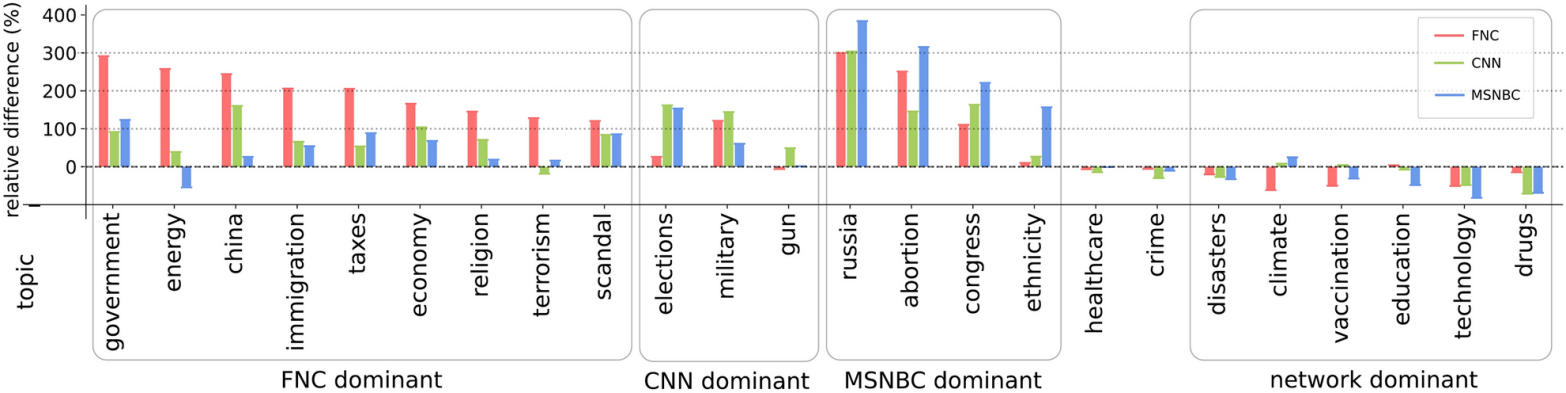
Relative differences in coverage between cable-FNC (red), CNN (green), and MSNBC (blue)-and broadcast stations, for the 24 topics. A positive value indicates that a cable station devoted a larger share of its coverage to a given topic relative to broadcast news.

To better understand how each topic contributes to these differences, we disaggregate the results at the topic level. Fig. 2. presents the relative differences in coverage for each topic *k*, $\delta ^{(i,j)_k} = 100 \times \frac{(y^{i}_k-y^{j}_k)}{y^{j}_k}$, representing how much cable coverage deviates from network coverage, where *i* refers to one of the cable stations and *j* refers to one of the network stations. (Similarly to Fig. 1, we use CBS as the reference station, but given the similarity between the broadcast networks, results do not change when CBS is replaced by NBC or ABC.) In some cases, the differences observed appear to reflect “partisan coverage filtering”^22^, wherein partisan cable channels preferentially cover issues that favor their viewers’ preferred political party^23^. For example, FNC’s disproportionate attention to the economy, taxes, immigration, and terrorism aligns with Republican politicians’ incentives. Similarly, MSNBC’s focus on abortion and ethnicity (which may evoke racially coded issues such as welfare and poverty) boosts political topics that benefit Democratic politicians. Partisan topic selection is especially apparent for salient political topics, with cable networks devoting a large share of their coverage to hot-button issues that resonate with their audience’s political predispositions. On the contrary, broadcast networks tend to lead in coverage of issues with less overt partisan coding: education, technology, and drugs.

More generally, surges in coverage of specific topics often map onto high-profile news events. Unpacking Fig. 2 over time, Fig. S6 shows the time series of the proportion of airtime devoted by the six stations to each topic. For example, the Russia topic tracks Russia’s potential involvement in the 2016 U.S. election and subsequent investigation, and then peaks again when Russia invaded Ukraine in 2022. The Supreme Court decision to overturn Roe v. Wade led to a spike in coverage of abortion across stations, yet MSNBC dedicated a far greater share of coverage to the topic. In another example, there is a spike in coverage of taxes in response to the 2017 Tax Cuts and Jobs Act (informally known as the Trump tax cuts), especially on FNC. The vaccination topic tracks with the introduction of the COVID-19 vaccine, where there is an increase in coverage of both vaccination and healthcare on broadcast network news, especially spiking in 2021. While both broadcast and cable news programming increased coverage of these topics in response to unfolding events, the sharp spikes diverge in both exact timing and size, leading to meaningful selection differences within these previously sleepy topics. An increase in polarization can be seen in coverage of the economy in 2017 during debates over the Trump administration’s economic policies (tariffs, tax cuts, etc.) and a spike over the 2020-2022 period coinciding with the COVID-19 pandemic’s economic impacts, where we observe FNC pulling away from other cable stations. Surges in immigration coverage appear in 2014 (reflecting debate over comprehensive immigration reform proposed by Obama), 2016-2019 (in response to border policies, asylum debates, etc., during Trump’s first term), and specifically on FNC in 2021 (following Biden’s reversal of several Trump-era immigration policies). Event-driven surges of coverage are not apparent for other topics, such as climate change, crime, and education. However, in general, fluctuations in topic coverage often reflect ongoing national politics and current events—especially presidential elections and changes in administrations.

Together, Figs. 1 and 2 show that viewers of broadcast news received largely interchangeable news regardless of which station they watched or when in time they watched it. In contrast, viewers of cable news saw a different mix of topics than those watching the broadcast networks, where these differences increased over time. FNC viewers saw more coverage of topics emphasized by Republican politicians (such as immigration and the economy), whereas CNN and MSNBC viewers watched news coverage that converged on topics from Democratic politicians’ agenda (such as abortion, ethnicity, and the Russia investigation). By 2022, FNC came to stand on its own as distinct from both the broadcast networks and the other two cable stations in how it allocates attention to topics (see Supplementary Materials, Figs. S9 and S10 as a robustness check for replication of results with a high recall topic inference model).

### Polarization in topic coverage

Although significant, the increasing discrepancy in topic selection potentially understates between-station differences in coverage. Even when two stations cover the same topic, they have considerable leeway to talk about it differently, in at least two ways. First, they may choose to cover distinct subtopics or aspects of the same topics, thereby focusing attention on different events, actors, or facts. For example, when discussing immigration, CNN and MSNBC may choose to cover the humanitarian issues associated with overcrowded camps of asylum seekers (e.g.,*“we should not be locking these people up in the way were doing it. craig, its atrocious. i think it goes against our values”* from MSNBC), whereas FNC may choose to cover border security or criminality (e.g., *“the border patrol apprehending thousands of migrants a day coming across all kinds of criminal activity as well as felons posing a threat to our safety”*). Second, two stations may cover the same subtopic of the same topic but use different linguistic framing to “spin” them in different ways. For example, within the subtopic of the death of George Floyd in May 2020, some commentators used linguistic frames about racial justice (e.g., *“it goes back so far, you know, all the way to the era of jim crow, people – black people walking down the street, being stopped by police officers for infractions like looking at someone the wrong way or addressing them the wrong way and being brutalized and incarcerated. and in this case, george floyd was killed in this interaction with police”* from CNN), whereas others used the language of partisan politics (*e.g., “i think ms pelosi is a little confused about who killed george floyd. but you know what, the cop that killed him was a democrat. the chief of police was a democrat, the mayor was a democrat, the city council was democrat. and you know what? the governor was a democrat”* on FNC).

Although these two mechanisms—selection on distinct subtopics and linguistic framing of the same subtopics—are theoretically distinct, in practice, the distinction is often subtle. For example, within the subtopic of vaccine safety, one station may emphasize the relative scarcity of adverse effects while another may focus on the tragedy of occasional deaths. However, at what point does a different choice of words to describe the same subtopic (a positive vs. negative spin on vaccine safety) morph into distinct subtopics (choosing to cover official vaccine safety statistics vs. detailed profiles of potential deaths due to vaccination)? Likewise, in the examples above, one could view the subtopics of basic humanity vs. potential criminality of asylum seekers as alternate framings of the same subtopic; and conversely, one could view the historical vs. political framing of George Floyd’s death as choices of different subtopics.

Given these practical constraints, we do not attempt to differentiate between subtopic selection and linguistic framing, instead operationalizing the difference in how the various stations cover news within a given topic in a way that captures both effects together. Specifically, we adopt a measure proposed by Gentzkow et al.^24^ to quantify the partisanship of congressional speech. In that context, the measure $\pi (x)$ corresponds to the average probability that a listener with no ex-ante information can correctly identify their political party (Democrat or Republican) after listening to a single two-word utterance (see Materials and Methods for details), and for our paper it will mean identifying a station. Thus, a value of 0.50 implies no discernible difference between the language used by the two parties, while higher values correspond to increasingly obvious differences. Historically, Gentzkow et al. found that their measure of partisanship fluctuated between 0.50 and 0.52 from 1870-1990 and then “exploded” in the 1990s and 2000s, reflecting a sharp rise in elite polarization that began with the Republican Party’s 1994 “Contract with America,” and reached a maximum of 0.54 in 2008, the year in which Barack Obama was elected president. In other words, the numerical difference between 0.52 and 0.54 for a single utterance is substantively significant, corresponding to posterior probabilities of 0.54 and 0.73, respectively, when the listener was exposed to as little as one minute of speech^24^. Thus, building on Gentzkow et al., we define “polarization” $\pi (x)$ in television news production as the average probability that a listener with no ex-ante information could correctly identify a given station after listening to a single one-word or two-word utterance.

**Fig. 3:**
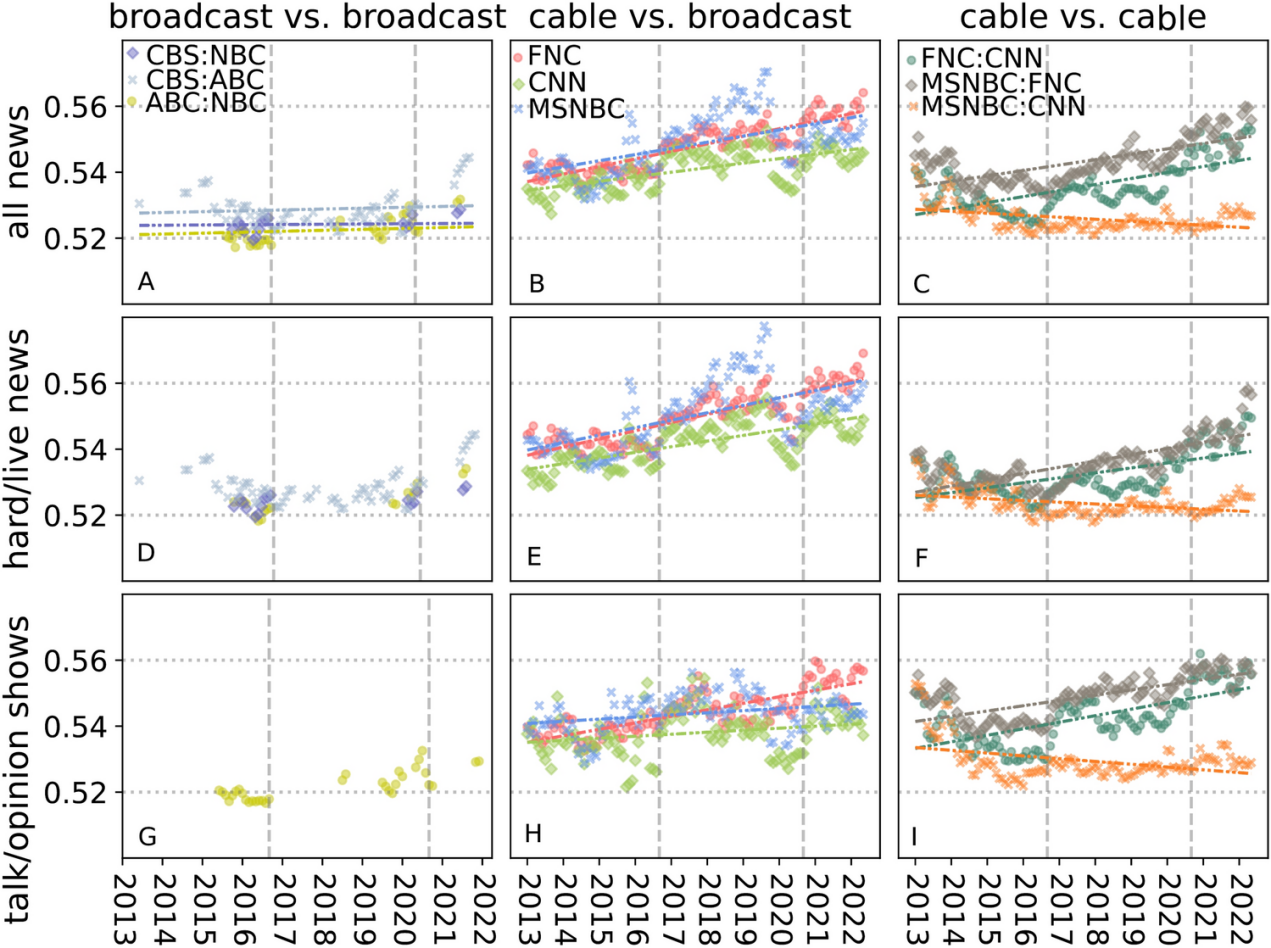
Averaged within-topic: (i) polarization between NBC vs. ABC (yellow), NBC vs. CBS (purple), and CBS vs. ABC (blue) over time for all news (**A**), hard news shows (**D**), and talk/opinion shows (**G**); (ii) polarization of the cable networks-FNC (red), CNN (green), and MSNBC (blue)-with respect to broadcast news over time for all news (**B**), hard news (**E**) and talk/opinion shows (**H**); (iii) within-cable polarization, comparing FNC with CNN (green) and MSNBC (grey), and CNN with MSNBC (orange) for all news (**C**), hard news (**F**) and talk/opinion shows (**I**).

#### Polarization over time

Figure 3 shows trends in polarization over time within and between broadcast and cable news networks for all news types (panels A-C), hard news only (panels D-F), and talk/opinion shows (panels G-I), which includes shows such as MSNBC’s Morning Joe (see Supplementary Materials for details on program classification). Referring initially to all news types, Fig. 3 (A-C) shows three main trends. First, Fig. 3A, which compares the broadcast networks with one another (NBC vs. ABC [yellow], NBC vs. CBS [purple], and CBS vs. ABC [blue]), shows that, similar to topic coverage (Fig. 1), polarization between broadcast networks is low and not increasing discernibly over time.

Second, Fig. 3B shows the polarization of the cable networks—FNC (red), CNN (green), and MSNBC (blue)—with respect to broadcast news, where to create our “broadcast news” baseline we first combined all utterances from NBC, CBS, and ABC (the results are not substantively different when we substitute with a single broadcast network; see Supplementary Materials for more sensitivity analysis, Figs. S11-S13). It is critical to note that this polarization is within topic, capturing differences in language conditional on talking about a given topic. Here we see a different picture: average polarization across all three stations starts around 0.54, already higher than the within-broadcast average, and then increases by approximately 0.016 over the study period, a change that is comparable to the shift from 0.52 to 0.54 that Gentzkow et al. characterized as “explosive” in the context of congressional speech. Moreover, the overall increase is mostly due to FNC, which starts higher (0.54) and increases by more (0.025) than the other two stations. Although apparently small in magnitude, these numerical differences correspond to highly salient differences in the language being used. To illustrate, consider the following low-polarization segments on the topic of Russian election interference from MSNBC and FNC, respectively: “...*in 30 minutes, the deputy attorney general and fbi director will testify about 2016 russian election hacking. *...” on (0.50); and “...*fbi director and the nsa chief heading back to capitol hill next week. to testify before the house intelligence committee. part of the intelligence into russian election meddling*” (0.48). Although these segments appeared on cable networks with opposite partisan leanings, the language used is indistinguishable; simply listening to the words, one would not be able to reliably say what the source was. In contrast, consider the following high-polarization segments, also on the topic of Russia and again from MSNBC and FNC respectively: “...*donald trump went bust. he could not borrow money from banks. it was coming from Russia, and once he’s out of office you’re going to see money laundering, you’re going to see a criminal enterprise*...,” (0.60); “...*there is no trump-russia collusion but hillary did pay for russian lies that was used to disseminate lies to the american people*...,” (0.65). In this case, the differences in language are stark and reflect clear partisan talking points; listening to these segments, one would have no trouble identifying which was from MSNBC and which was from FNC.

Overall, we see a gradual increase in polarization of cable news compared to broadcast in the observation period, Fig. 2B. As with topic selection (see Fig. S6), there are also peaks and drops that correspond with major events. For example, increases in polarization coincide with the last two presidential elections in our sampled timeframe: one spike in polarization beginning in the fall of 2016 across all cable stations and another in the fall of 2020. After each spike, the polarization scores typically decline modestly but remain on a higher plateau than before, suggesting that polarization levels now have a higher baseline than observed in previous years. Another pattern that emerges here is that among the three cable stations, MSNBC had the highest polarization on average during Trump’s first term, whereas FNC had the highest polarization during Biden’s presidency. Unfolding major events do not necessarily lead to increases in polarization. For example, the temporary drop in polarization in early 2020 coincides with the early stage of the COVID-19 pandemic. News coverage became more homogeneous across TV stations as news programs focused their coverage on breaking news related to the pandemic and response efforts. Polarization rose again in late 2020 as the news agenda changed, with breaking news coverage of the pandemic giving way to topics such as debates over public health policies (e.g., mask mandates and lockdowns) and the 2020 presidential election, later reaching a peak around the January 6th Capitol Riot in early 2021.

**Fig. 4:**
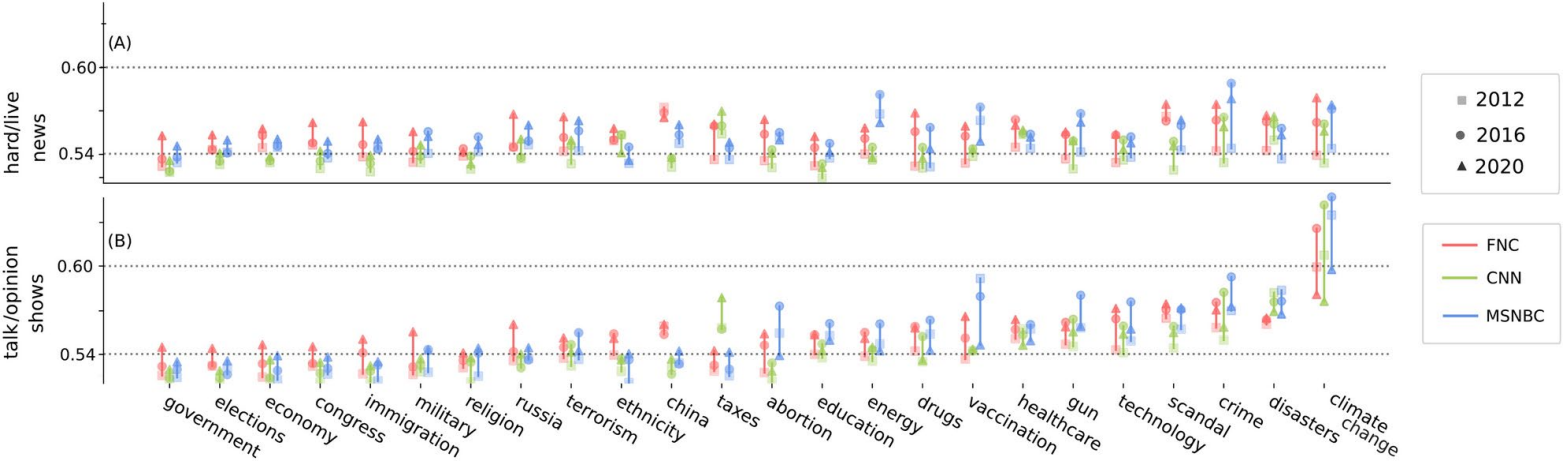
Topic-level polarization for FNC, CNN, and MSNBC for talk/opinion shows (**A**) and hard/live news shows (**B**) compared with broadcast news networks. On the left side, we see the least polarized topics. Here, the highest levels of partisanship observed among these topics appears in 2020. As we move toward the right, partisanship increases. For these topics, the highest levels of polarization are generally during Trump’s first presidency.

Third, Fig. 3C shows within-cable polarization, comparing FNC with CNN (green) and MSNBC (grey). Crucially, at the beginning of the time period (in 2013) polarization between FNC and both CNN and MSNBC is not perceptibly different than it is between CNN and MSNBC themselves, and the overall level is comparable with polarization within broadcast networks. By 2023, however, FNC has diverged strikingly from the other two, while MSNBC and CNN have become less polarized relative to one another. In other words, while FNC covered a different mix of topics than CNN and MSNBC in 2013, when they did cover the same topic they used similar language. By 2023, however, FNC had not only departed from MSNBC and CNN in terms of the mix of topics; they were also covering the same topic differently.

The remainder of Fig. 3 shows that these three trends—low and not increasing polarization for broadcast networks, higher and increasing polarization between cable and broadcast, and divergence between FNC and the other two cable networks—also apply separately to hard news (panels D-F) and talk/opinion shows (panels G-I). In addition, we make two further observations. First, the increase in polarization between cable and broadcast news that is apparent in Fig. 3B is driven more by increased polarization among hard news programming (Fig. 3E), than by opinion/talk shows (Fig. 3H). Indeed, while hard news and talk/opinion shows exhibited roughly the same polarization in 2013, hard news was noticeably more polarized than talk/opinion shows by 2023, largely on account of a sharp jump that occurred in 2016. Second, within cable networks, a somewhat opposite result applies: in this case talk/opinion shows contribute more to the overall increase—again, driven by a sharp increase in 2016—and are more polarized overall than hard news programs.

#### Topic-level polarization

Moving beyond these station-level trends over time, Fig. 4 shows average polarization by topic, program type, and year (3 time periods of before 2016 [2012], 2016-2020 [2016] and after 2016 [2020]). Fig. 4A shows topic-level polarization for hard news/coverage of live events for FNC (red), CNN (green), and MSNBC (blue) versus broadcast news in those topics (as before, we compare individual cable news networks with an aggregation of the broadcast networks’ news content) and Fig. 4B shows the same for talk/opinion shows. In both cases, Fig. 4 shows considerable variation across topics. Starting with hard news (Fig. 4A), topics such as government and the economy start at relatively low values of polarization and increase by relatively small amounts over the time period, whereas other topics such as crime and climate change, jump to much higher levels in 2016 and 2020. For example, FNC’s least polarized discussions of climate change, e.g., “...*vice president al gore on ‘meet the press’ not mincing his words about the severity of the global climate crisis and america’s inaction*...” (0.48) are indistinguishable from broadcast news (again covering the same sub-topics with neutral linguistic frames), whereas its most polarized discussions, e.g., “...*the real cause of islamic terror is global warming. can you agree with that? no, i can’t, but that’s apparently what the president authentically believes*...” (0.70) are highly distinctive in both the unique sub-topic and incendiary linguistic frame. For talk/opinion shows, meanwhile (Fig. 4B), the differences across topics are even more stark. On the one hand, the low polarization topics demonstrate a similar pattern to their hard news counterparts, starting off low and not increasing by much. On the other hand, the high polarization topics start off more polarized and increase by even larger amounts (e.g., Tucker Carlson on climate change: “...*that screeching moron ocasio-cortez isn’t telling china to give up coal and oil and natural gas and nuclear power within a decade. the left isn’t doing any of that because the left loves the Chinese government. it’s their model for governing*...” [0.75]).

In other words, while the overall increase in polarization is driven more by the increase in hard news (see Fig. 3E), the most polarizing coverage still appears on talk/opinion shows where it is concentrated in a few extremely polarized certain topics.

**Fig. 5:**
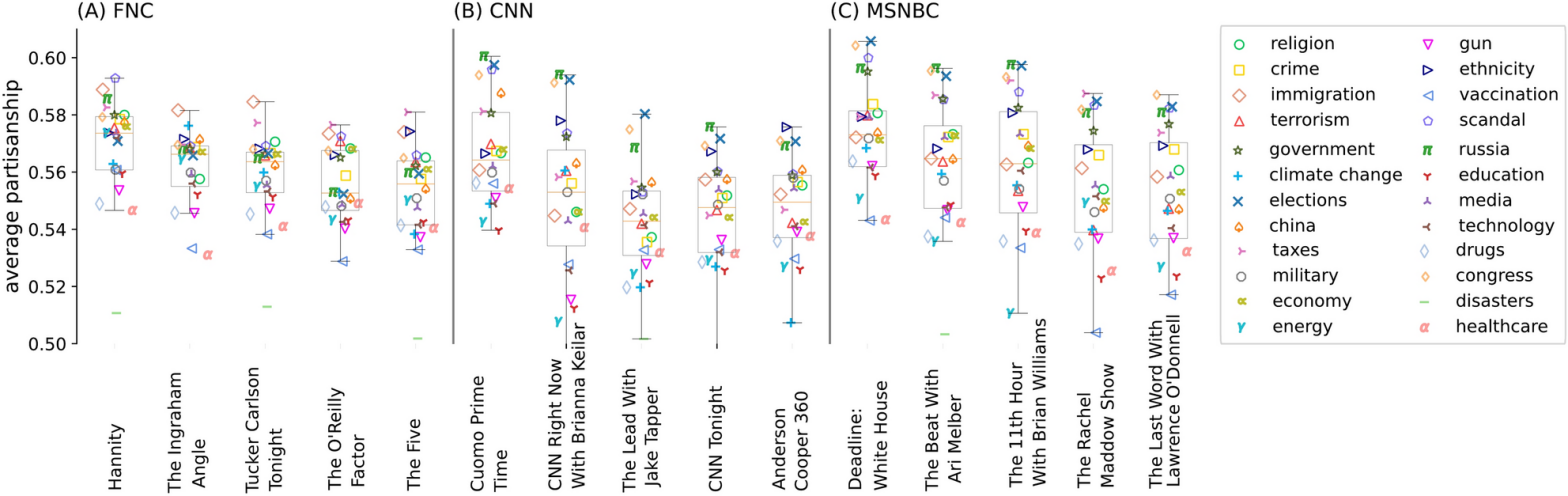
Boxplot of partisan scores at the program level. Markers show the average partisan score of each topic within that specific program.

#### Program-level polarization

Shedding more light on this last point, Fig. 5 shows polarization of the top five most popular programs identified by Kim et al.^20^ for FNC (Fig. 5A), CNN (Fig. 5B), and MSNBC (Fig. 5C) broken out by topic (again versus all broadcast news in the topic). Overall FNC has the highest number of highly polarizing shows—notably Hannity, The O’Reilly Factor, Tucker Carlson Tonight, and the Laura Ingraham Angle—however, a handful of MSNBC shows, led by Deadline: White House (with Nicolle Wallace), are equally polarized. CNN’s leading talk/opinion shows tend to be less polarized than those on FNC or MSNBC, yet Cuomo Prime Time stands out as one of the most polarized programs across all three networks.

Beyond network-wide differences, certain topics drive heightened polarization within programs. FNC popular shows are more polarized on topics such as immigration and taxes, while CNN and MSNBC popular shows are most polarized on elections, Russia, and Congress. For example, Hannity features highly polarized language when discussing immigration (“...*some democrats even proposed a measure that would force the release of thousands of criminal illegal aliens, including dangerous felons convicted of rape, sex trafficking, violent assault, and even murder into our country. can you believe it?*...” [0.62]) whereas Deadline: White House uses the most polarized language around elections (“...*the demise of democracy is happening in full view, predicated on a lie, designed to make it harder for people to vote and to politicize the people who count the votes*...” [0.63].)

**Fig. 6:**
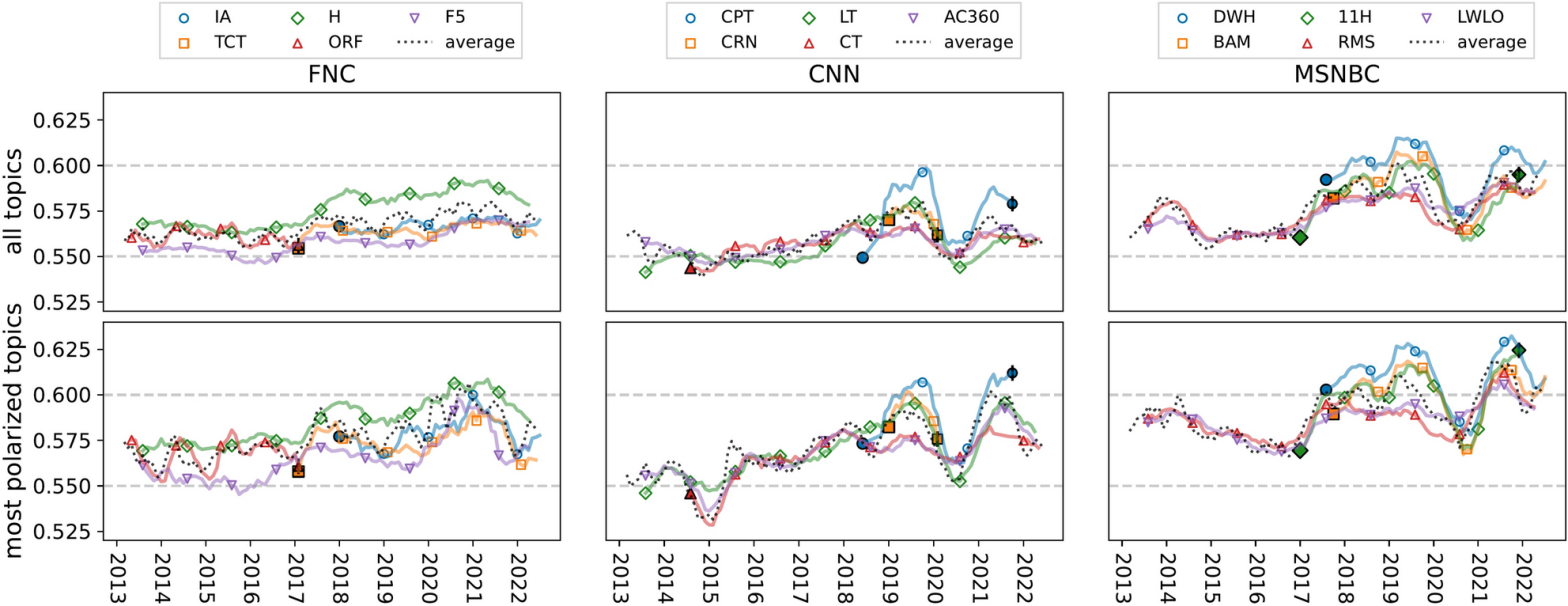
Time series of program-level polarization for FNC, CNN, and MSNBC, averaged over all topics (top row) and a select set of highly polarized topics (bottom row), over a select set of five programs from each station. Selected topics include: immigration, taxes, Russia, and scandal for FNC; congress, scandal, Russia, elections for CNN; and congress, taxes, Russia, and elections for MSNBC. Dotted black lines represent the average polarization of the five networks for each network. Filled markers indicated the start and end (with the vertical line) date of the shows. Abbreviations for programs: FNC: IA (The Ingraham Angle), H (Hannity), TCT (Tucker Carlson Tonight), ORF (The O’Reilly Factor), F5 (The Five); CNN: CPT (Cuomo Prime Time), CRN (CNN Right Now With Brianna Keilar), LT (The Lead With Jake Tapper), CT (CNN Tonight), AC360 (Anderson Cooper 360); MSNBC: DWH (Deadline: White House), BAM (The Beat With Ari Melber), 11H (The 11th Hour With Brian Williams), RMS (The Rachel Maddow Show), LWLO (The Last Word With Lawrence O’Donnell).

While these patterns illustrate the issue-level polarization of specific programs, they do not capture how program-level polarization has evolved over time. Figure 6 presents the time series of polarization scores for selected programs, highlighting trends in the stability of polarization within programs over time. Focusing on the top row, we observe that the rapid increase in polarization beginning around 2016 coincides with the introduction of new cable news programs with high polarization scores and that there is significant heterogeneity in within-program polarization trends. For example, Tucker Carlson Tonight and The Ingraham Angle on FNC have high, stable polarization levels from their introductions in 2016 and 2017 respectively. In contrast, Cuomo Prime Time and CNN Right Now With Brianna Keilar experience large fluctuations during their relatively short runs on CNN. A similar pattern is observed on MSNBC for Deadline: White House, The Beat with Ari Melber, and The 11th Hour With Brian Williams. The spike in polarization in 2016 also reflects existing programs with previously stable polarization scores adopting more polarized language around this time. On FNC, Hannity and The Five exhibit a slow but steady rise, with a pronounced jump in 2016. On CNN, Anderson Cooper 360, The Lead With Jake Tapper, and CNN Tonight maintain a consistent presence with a moderate increase in polarization. On MSNBC, The Rachel Maddow Show and The Last Word with Lawrence O’Donnell display cyclical patterns across administrations, with both gradually becoming more polarized. All these patterns are more pronounced among the top four most polarized topics for each station (bottom row).

These findings underscore the temporal dynamics of polarization at the program level. In general, we do not observe a systematic association between how long a program aired for and its polarization level (Fig. S8 A). Instead, polarization patterns vary by topic and network. For example, for discussion of taxes, polarization is higher for short-lived programs on FNC, while CNN exhibits the opposite trend, and MSNBC shows no clear association in this topic (Fig. S8 B). In contrast, for coverage of Russia, CNN, and MSNBC programs that have been on air longer tend to be more polarized, whereas FNC shows no significant relationship between program longevity and polarization (Fig. S8 C).

#### Qualitative examples

To facilitate the interpretation of the polarization measure, we first provide qualitative examples from news transcripts. These examples show how different stations’ coverage of the same narrative—here, Robert Mueller’s congressional testimony and the Trump administration’s family separation policy—can have either high or low polarization scores depending on the distinctiveness of the language.

The media frenzy around the Mueller investigation into collusion between the 2016 Trump campaign and the Russian government reached a high point in July 2019 when Robert Mueller presented his findings to Congress. High-polarization segments often reflected partisan talking points on both FNC (e.g., “...*there is no trump-russia collusion but hillary did pay for russian lies that was used to disseminate lies to the american people*...,” polarization score of 0.65) and MSNBC (“... *donald trump went bust. he could not borrow money from banks. it was coming from russia. and once hes out of office, youre going to see money laundering. youre going to see a criminal enterprise...*,” [0.60]). In contrast, low-polarization segments often employed similar language to describe ongoing events, evident across both MSNBC (“...*a previously undisclosed encounter between the president and attorney general jeff sessions in march, 2017 where the president urged sessions not to the recuse himself from the russia investigation...*” [0.50]), FNC (“... *last night, he went after jeff sessions saying he was angry that sessions had recused himself from the russia investigation and if he ‘had to do it over again,’ he ‘would have hired someone else’ to be the ag*...” [0.48]), and other stations. On FNC, viewers saw coverage that characterized the investigation as a “smear job” and a “hoax”; emphasized John Durham, who was been appointed to investigate the FBI’s handling of the Trump-Russia case; and voiced allegations that the FBI had illegally spied on the Trump campaign. On CNN, viewers saw coverage that scrutinized the Mueller hearings, providing fuel to claims that Trump had obstructed justice; opined about whether Trump may face legal liability; and deliberated U.S. foreign policy towards Russia. Russia segments with low polarization scores—those without distinctive FNC, MSNBC, or CNN language—focused on the impact of the Russia scandal on Trump’s polling and summaries of Mueller’s primary findings.

In June 2018, the U.S. government acknowledged that it had been separating migrant children from their families. On FNC, segments high in polarization focused on topics such as the professionalism and efficiency of the U.S. immigration officials, the potential for new federal immigration policy under Trump, and the Supreme Court’s decision to uphold the travel ban. For instance, a high-polarization FNC segment distanced individual border patrol agents from the policies they enforced (“*... i think the agency could conceivably be split because the backlash for that horrible family separation is not just among civilians. its even among the agents themselves...*” [0.60]). CNN’s high-polarization immigration segments, however, described a humanitarian crisis in central America contributing to migration, characterized family separation as inhumane, and emphasized stories about young children taken from their parents (e.g., “*...clearly no plan to roll it back, right. thats why you still have 3,000 kids without their parents...*” [0.59]). In contrast, low-polarization segments summarized recent developments on both CNN (“*...officials say nearly 250 immigrant children remain in usa custody separated from families...*” [0.43]) and FNC (“*...the justice department’s watchdog releasing a report finding former attorney general jeff sessions and other top doj officials were the driving force behind family separations at the border back in 2018...*” [0.47]). See Supplementary Materials, Table S4, for more examples.

## Discussion

Motivated by concerns about partisan-segregated television consumption^18,19,22^, we conducted a computational content analysis of TV news on both cable and broadcast stations covering a ten-year period. We find that bias has become more pronounced over time in TV news: The three major cable news channels are increasingly polarized relative to the broadcast networks, covering different topics and using increasingly different language to talk about them^25–27^. Within cable, we also find that FNC has diverged increasingly from CNN and MSNBC, which have simultaneously converged. Surprisingly, we find that these differences appear to be driven more by the increase in polarization of hard news programming than by talk/opinion shows, although the latter appear to host the most polarizing content. These results reinforce conventional wisdom and scales previous work^20,22^ on the polarizing effects of cable news to the majority of segments on cable across nearly 10 years while adding contrasting with broadcast news, the dominant source of national TV news.

The limited analyses of media bias that focuses on production, rather than consumption, generally measure bias in television news as either linguistic similarities to partisan sources^28^ or by tracking topic selection^19^. On its own, either of these approaches leaves considerable ambiguity: linguistic bias could reflect topic selection, the language used to talk about those topics, or both, and bias in topic selection may fail to capture a critical source of bias if two stations’ coverage of the same topic takes different forms. Different forms of bias have different implications for viewers^29^. Here, we disaggregate these forms of bias and show that both have become more prevalent in cable news in recent years. While partisan news has employed us-versus-them frames in political coverage for decades^30^, the recent divergence in cable networks’ topic selection and framing (see Figs. 2 and  1) seems likely to amplify its polarizing effects. As partisan bias has become more prevalent in cable news production, one-channel cable news viewers are increasingly exposed to a set of facts, narratives, and arguments that have been curated with a consistent partisan goal and lack counterarguments^30^, all of which is designed to prime their audiences to approve of the conduct of favored politicians and disapprove of opponents. At the same time, we also find that the “big three” broadcast networks (ABC, CBS, and NBC) are largely interchangeable, covering a similar mix of topics and talking about them in a similarly neutral manner and that these similarities are stable. To the extent that Americans get their news from the “big three” broadcast networks, they likely do experience a common sense of understanding about current events, whereas to the extent they segregate themselves by cable channels, they likely do not.

We contextualize and augment these findings about the production of news with insights on consumption on the consumption of news by replicating the results of Ref.^18^ on U.S. adults’ cable news consumption and extending the analysis to encompass recent years (to align with our production study period) and provide a side-by-side comparison of broadcast network consumption trends (see Supplementary Materials, Section D, for more details). We find that while, on the production side, broadcast news remains a source of coherent content, its consumption has declined dramatically: focusing on Fig. S14 (C), in 2016 about 35% of U.S. adults primarily consumed broadcast news, that number was down to around 25% by 2023. And, the growing polarization of cable news coincides with stability in the share of U.S. adults that consume primarily cable news (about 15%). The steady consumption of fragmented sources, coupled with the decline in the consumption of common sources, amplifies concerns that Americans are increasingly losing a common understanding of events through TV.

In closing, we emphasize that the ultimate significance of an increasingly biased media environment—and whether the situation is as dire as former President Obama suggested—is an ongoing research debate that we inform but do not solve in this paper. On the one hand, to the extent that a common factual baseline in news media does facilitate social integration, as we suggested in our opening motivation, then it follows that observed increases in partisan polarization^28,30,31^, may indeed be due in part to the resulting loss of information diversity and lack of shared understanding as a result of citizens who opt out of consuming news altogether or choose to consume their news from distinct sources that offer increasingly incommensurate views of the world. A large and growing body of research suggests that certain features of news content—such as the topics covered and the narratives, facts, and frames presented in an outlet’s coverage—do affect viewers’ beliefs, feelings, and behaviors. For example, news media can influence viewers by shaping how they assess the importance of political issues^21,32,33^, formulate policy opinions^22^, talk about politics^34^, and evaluate politicians’ performance^23^.

On the other hand, we note that the media’s contribution to polarization is contested^35^ and may be overstated relative to other hypothesized causes, such as the polarization of political elites^36,37^, economic inequality^38^, and long-run social and demographic changes^39^. And, evidence of the media’s potential to polarize viewers at the individual level cannot support claims about population-level change. For instance, although there is evidence of a causal pathway between watching partisan television and adopting more extreme policy positions^5^, it does not follow that partisan television caused mass polarization. The aggregate effect of partisan news will be small if partisan news viewers also encounter countervailing perspectives or if few citizens consume polarizing news content in the first place. Even the most resource-intensive, large-scale field tests of media effects^19^ cannot demonstrate if news media shifted opinions and behaviors at the population level. Estimating the aggregate impact of news media necessitates measuring who is consuming what.

In addition, we note three other limitations of our analysis that we hope will motivate future work. First, while our contribution advances on previous work in that it separately accounts for differences in topic selection and what we call polarization, our polarization metric does not further separate out differential emphasis on subtopics from linguistic frames. Because differential emphasis on distinct elements of a topic could potentially have different implications for viewers’ perceptions than differences in the language used to describe the same elements, an outstanding challenge for future work is to develop scalable methods for reliably making this distinction. Second, we have only studied English-language television programming, a restriction that omits several popular Spanish-only networks such as Univision and Telemundo, which collectively account for roughly 5% of the overall audience for television news. Lastly, while our work offers new insights by stratifying news across topics, program types, and individual programs, it is purely descriptive and does not establish the causes of the growing divergence between cable networks and broadcast news. A thorough understanding of the factors—such as major events, addition/removal of programs, or shifts in station business models—driving these changes in TV news production requires careful study design and control over potential confounders, and this remains an important direction for future research.

## Materials and Methods

### Data

Our dataset, sourced from TVEyes, a media monitoring firm, comprises transcripts of all programs aired by six major U.S. television networks: FNC, CNN, MSNBC, ABC, CBS, and NBC. In this paper, we focus exclusively on news programming, which encompasses 328,432 episodes broadcast between December 2012 and October 2022. For ABC, CBS, and NBC, we analyze nationally syndicated news programs and do not analyze any local, market-specific news programming. These data represent the population of national TV news across these six channels for the observed period (see Table S1 and Supplementary Materials, Section A).

### Text classification

As a first step, we remove all the advertisements in our entire transcript data set. Since each episode of a news program may cover multiple topics, we split each ad-free episode transcript into “segments” that can contain up to 150 words, resulting in a total of 13, 446, 736 segments across all six stations (see Supplementary Materials, “text cleaning” section for more information). The next step is identifying topics discussed in each segment. While topic models can be very effective at uncovering topics within large collections of documents, scholars have expressed concern about measuring concepts using topic models without assessing their accuracy^40,41^. To rigorously quantify how major TV stations cover important topics of the day, we propose a novel two-layered human-in-the-loop multilabel classification model, where a segment can belong to none, one, or multiple of the predefined labels.

Because we classify segments across a wide range of topical dimensions, traditional approaches to supervised classification would be infeasible. Training and validating a multilabel model or a series of binary classifiers would also require extensive annotated data, which would be inefficient and costly: (i) it would require annotators to select a topic from a large list of candidates, (ii) considering that class imbalance varies across topics (and is likely to be high for the majority), it would be necessary to sample approximately 10,000 segments for a topic covered for less than 5 percent of the total news airtime in order to collect 500 positive results. Instead, we propose a two-layer human-in-the-loop approach whereby, in the first layer, a weakly-supervised classifier with high recall is utilized to narrow the search space for segments belonging to each topic. The objective of this layer is to identify samples that are highly likely to be positive for each class on the basis of as little information as one word per topic. The first layer produces a set of candidate segments for each topic that are high in recall but low in precision. By removing irrelevant segments from consideration for each topic, the first layer greatly increases the true positive ratio for each topic, improving class imbalance. As a result, supervised classification performed in the second layer is more efficient. Within each topic, we select a random set of segments for annotation from the reduced set, then ask an annotator to select from a limited number of three topics, including the one that has the highest probability. The annotator can indicate that a segment should be classified as none or one of the provided topics. With the human-labeled segments as our ground truth, we develop supervised models in the second layer to refine low-precision classifications from the first layer. Fig. S2 provides an overview of our pipeline for segment classification, including the occurrence of each label.

### Weakly-supervised layer

Keyword matching is one of the common methods used to classify documents into a set of predefined topics. It is, however, limited by low recall—(i) there are several (or many) keywords that may be representative of a topic, (ii) a topic may be discussed without the use of common keywords—and low precision—(i) the use of a topic-relevant word does not necessarily indicate it is the topic of the document; (ii) the same word can have a variety of meanings (for example, lead poisoning vs. lead officer). To overcome these shortcomings, we develop an “expanded dictionary” for each topic containing words semantically similar to its corresponding label word, as proposed by Meng et al.^42^ (It should be noted that the label names used to refer to topics are to some extent arbitrary and, for a better understanding of what each topic represents, the category vocabulary lists are included in the Supplementary Materials, Table S7). Then, each segment is assigned to a topic if it contains words semantically similar to those in the topic’s expanded dictionary. This approach assumes that, inherently, words with similar meanings are interchangeable. Thus, to create an expanded dictionary for each topic, we identify a suite of words that are semantically close to the topic label. To populate each expanded dictionary—by finding the most likely words that would replace each topic label in context—we use a pre-trained masked language model (MLM)^43^. For each topic, we successively mask each occurrence of the label word in the corpus and then feed the resulting contextualized embedding vectors from the BERT encoder to the MLM head. The output is a probability distribution over the entire vocabulary *V*, which indicates the likelihood of each word *w* appearing at the given position where the topic label word was masked. Our system loops over each segment and stores the top 50 probable replacements for each label occurrence, Algorithm S1. Next, for each topic, we rank the candidate words by the number of times they appear in a top-50 most probable replacements list and create a “class vocabulary” set for each topic. To do so, we keep the top 100 most frequent replacement words. After creating such a vocabulary for each topic, the words within each set are manually reviewed by authors, and the ones that are too general or irrelevant to the label of interest are removed. The resulting list for each topic comprises our expanded keywords.

By utilizing the developed class vocabulary, each word in the corpus is examined within its context. Each document *d* is subjected to the following process: (1) mask each word *w* in document *d*, (2) for each masked word *w*, predict the top 50 words that can replace it, (3) determine the overlap between the predicted set for word *w* and class vocabulary for label *z*, and (4) assign the corresponding topic to document *d* when the overlap exceeds a predefined threshold for at least one word in the document^42^. Each segment passes through this layer and obtains a label per topic that indicates that it either belongs or does not belong to the corresponding class label. Consequently, each segment may be associated with multiple topics. It is important to note that a high threshold creates a more stringent limit for topic assignment, which may result in greater precision at the risk of losing true positives, and vice versa for a low threshold. Because the first layer optimizes for recall, we set a lenient threshold of 20% overlap, which results in a greater number of false positives. This first layer greatly reduces the search space for each topic *z* and improves its class imbalance (from ${\mathcal {D}}$ to ${\mathcal {D}}^{\text {weak-sup}}_z$), resulting in a more efficient second-stage annotation process.

### Topic annotation

We proceed by selecting 50 random segments from each topic-station, for a total of 300 segments per topic *z* from the corresponding set ${\mathcal {D}}^{\text {weak-sup}}_z$. Using the Amazon Mechanical Turk crowdsourcing service, four annotators label each segment—where we break ties with more annotations—summing to a total of almost 30 thousand annotations (see Supplementary Materials for more information, Fig. S1). A majority vote is required for the assignment of a topic to a segment. The labeled data is subsequently used as ground truth to train and validate supervised classifiers in the second layer. We also evaluate the precision of first-layer predictions, which is a weakly-supervised model where we only feed the class label names into the model, Fig S4.

### Supervised classification layer

The inputs for the second layer are set ${\mathcal {D}}^{\text {weak-sup}}_{(\text {station},z)}$ and the ground truth obtained from human annotators. For each station-topic pair, we perform 5-fold cross-validation in the training phase and choose the model with the highest F1 score (see Supplementary Materials for more information on model selection). Then, passing all the segments in set ${\mathcal {D}}^{\text {weak-sup}}_{(\text {station},z)}$ through its corresponding trained model, we create a set containing segments with the positive class label as ${\mathcal {D}}^{\text {sup}}_{(\text {station},z)}$. We achieve an average precision of $78.45\% \pm 13.65$ over $24\times 6$ models (see Supplementary Materials for more details on proposed Algorithm S1 and model performance, Figs. S4-S5). All the results in the main text are based on the output of the second layer (${\mathcal {D}}^{\text {sup}}_{(\text {station},z)}$), which achieves far higher precision than the outputs from the first, weak-supervised layer alone. As a robustness check, we also provide the selection and polarization results over the output of the first layer (${\mathcal {D}}^{\text {weak-sup}}_{(\text {station},z)}$), which has higher recall, Figs. S9-S11).

### Measuring polarization

Following Refs.^24^ and^44^, which study partisanship within congressional speeches and social media polarization respectively, we define polarization within our study as the probability that an observer with a neutral prior will assign a segment to its true station after observing a unigram or bigram from that segment. When considering a given representation *x* of segment *d*, we define the degree of polarization as the divergence between $x^{target} (d)$ and $x^{source} (d)$. When these vectors are close in the representation space, this indicates that the source and target speak in a similar manner, and we refer to this as polarization is low. When the language used by the source and target are more distinct, the vectors will be more distant, indicating higher polarization. To measure the partisanship between two stations, we use the leave-out estimator from Gentzkow et al.^24^, defined as:

1 $$\begin{aligned} {\hat{\pi }}^{LO} = \frac{1}{2}\sum _{i\in source} {\hat{q}}_i . \hat{\rho _{-i}} + \frac{1}{2}\sum _{i\in target} {\hat{q}}_i . (1 - \hat{\rho _{-i}}) \end{aligned}$$

For segment representation, we use the vector of unigrams and bigrams (phrases) frequencies, where ${\hat{q}}_i = {{\textbf {c}}}_{i}/m_i$, ${{\textbf {c}}}_{i}$ is the vector of phrase counts and $m_i=\sum c_{ij}$ is the total number of phrases in segment *i*. Note that equation 1 is symmetrical and there is no order to source and target stations. Furthermore, we remove possible confounders, e.g., for a given station we remove program names, hosts’ names, and mentions of the station name itself. Let ${\hat{q}}^{G} = \sum \limits _{i\in G} \hat{{{\textbf {c}}}}_i / \sum \limits _{i\in G} {\hat{m}}_i$ is the empirical term frequencies for station *G* and ${\hat{\rho }} = {\hat{q}}^{source} \oslash ({\hat{q}}^{source} + {\hat{q}}^{target})$, where $\oslash$ denotes element-wise division. Then, $\hat{\rho _{-i}}$ is the analogue of ${\hat{\rho }}$ computed from the empirical frequencies where segment *i* is excluded. The segment-level estimate of the partisan score for segment *i* is also defined as the dot product ${\hat{q}}_i \cdot {\hat{\rho }}_{-i}$^44^.

## Electronic supplementary material

Below is the link to the electronic supplementary material.

Supplementary Information.

## Data Availability

The data are made available to us by third companies under agreements with the University of Pennsylvania that prohibit sharing with any third parties without their prior consent. Interested parties should contact the corresponding author for further information.
