# Supplementary material for: Unpacking media bias in the growing divide between cable and network news
Source: Sci Rep. 2025 May 21;15:17607. doi: 10.1038/s41598-025-01046-7 (PMC12095647; doi:10.1038/s41598-025-01046-7)
Supplement: Supplementary file 1 — Supplementary Information. [file 41598_2025_1046_MOESM1_ESM.pdf]

Supplementary Information
Unpacking Media Bias in the Growing Divide Between Cable and Network News

Homa Hosseinmardi[1,2*], **Samuel Wolken**[2], **David M. Rothschild**[3], **and Duncan J. Watts**[2,*]

[1]University of California Los Angeles, CA, 90024 USA
[2]University of Pennsylvania, Philadelphia, PA 19104, USA
[3]Microsoft Research New York, New York, NY 10012, USA
[*]homahm@ucla.edu, djwatts@seas.upenn.edu

ABSTRACT

The potential for a large, diverse population to coexist peacefully is thought to depend on the existence of a public sphere in which citizens are exposed to similar facts about similar topics. A generation ago, broadcast television news was widely considered to serve this function; however, since the rise of cable news in the 1990s, critics and scholars have worried that the corresponding fragmentation and segregation of audiences has caused this baseline of common understanding to be lost. Recent work documents that millions of Americans are loyal consumers of cable TV news stations. However, the implications of partisan segregation in TV news consumption depend on bias in content—which topics TV news programs talk about and the language theye to talk about them. Here, we measure bias in the production of TV news at scale by analyzing nearly a decade of TV news (Dec. 2012–Oct. 2022) on the largest cable and broadcast stations. We quantify the share of attention each station devoted to more than 20 politically significant topics as well as the linguistic similarity of different stations' news coverage of those topics. We find that while broadcast news continues to cover similar topics with similar language, cable news stations have become increasingly distinct, both from broadcast news and from each other, diverging in terms of both content and language. This trend is driven by hard news as much as partisan commentary programs. Our results show that changes in the supply, not just consumption, of TV news are contributing to Americans' polarizing media diets.

Supplementary Information

A Program categorization

The selection and framing of topics by news networks reflects both editorial decisions made by the staff of individual news programs, as well as decisions made by networks about what sorts of programs to air. If the selection and framing of topics by talk shows and hard news programs on a particular cable station stay consistent, but the network opts to devote more airtime to talk shows than hard news, the station's news content will change in aggregate without change in the editorial judgment of any individual programs.

To analyze the network-level trends in airtime devoted to various types of shows, we categorized television news programs that aired on ABC, CBS, NBC, CNN, FNC, and MSNBC into the following categories: hard news, talk show, partisan/opinion news, soft news, local news, other. Table S1 shows the average news content per day per station. News programming for broadcast networks is based on affiliates in the New York, NY media market (WABC, WCBS, WNBC). The annotation process involved two undergraduate research assistants reviewing the name of each distinct news program that aired on one of the six channels during the study time period (see Table S2 for a description of each category). As this analysis specifically describes television news, we labeled only those television programs categorized as "news" by the vendor from which we acquired transcripts. The undergraduate coders had been instructed on the characteristics of programs from each category and went through a training period in which their annotations were calibrated to a gold standard. Each program was labeled by the two coders independently, and later on they were instructed to discuss the subset of programs they had disagreed on and come to a consensus. Afterwards, a Ph.D. student reviewed a sample of programs for quality assurance.

In the main text, for ease of interpretation, we aggregated the program labels into two main categories: a) hard news and coverage of live events; b) talk shows and partisan/opinion news, soft news.

B Topic selection

To generate a list of topics for classifying television news coverage, we began by reviewing responses to Gallup's "most important problem facing the country" survey item. We aggregated responses to this question over an approximately 13-year time frame: October 2008 to January 2022. Gallup poses this as an open-ended question, but responses are grouped into categories (such as "unemployment/jobs," "cost of living/inflation," and "dishonesty/lack of integrity") in Gallup's public-use data. The combined list of topics, spanning the entire time frame, included 366 different categories; however, after accounting for varying punctuation, misspellings, or slight variations in wording, we were left with a preliminary list of approximately 80 categories. We manually reviewed these categories and grouped them under high-level topics (e.g., grouping "inflation" and "unemployment/jobs" under "economy"). This process produced a list of 18 higher-level topics. We subsequently reviewed a random sample of national television news transcripts to identify any recurrent topics that were not covered by the topics generated from Gallup data. Based on this review, we added several additional categories to our list. Next, we supplemented our list of categories by identifying segments in the transcripts that would fall under each topic and summarizing any relevant topical focus. Our final list included 30 potential topics. Finally, we produced keywords for each higher-level topic that would be used to classify segments. In the main text, we present the results for 24 topics for which we had reliable models, Table S7.

C Segment classification

There are two general approaches to handle classifying large-scale unlabeled data when a subset of categories or labels is known: (i) weak supervision, and (ii) seed-based topic modeling. When there is no ground truth for classifying text, zero-shot learning methods are also very effective[1]. In spite of this, we found using the existing pre-trained packages to be exceedingly slow, due to the large number of topics considered in our classification task[2].

Table S1. Average news content per day per station. News programming for broadcast networks is based on affiliates in the New York, NY media market (WABC, WCBS, WNBC).

Station	Duration
CNN	19.99
FNC	22.80
MSNBC	22.50
ABC	11.09
CBS	8.62
NBC	10.24

As we prioritized validating our classification model performance, we chose a supervised approach rather than topic modeling. With 13 million data points, annotating enough samples to perform a fully supervised classification with 30 target variables is very costly. In order to categorize text documents, we chose a weakly-supervised approach in which only word-level descriptions are used, Algorithm S1-S2. The performance of our model was then evaluated using a small collection of annotated holdout documents. Using the Amazon Mechanical Turk crowdsourcing service, four annotators labeled each segment—where we break ties with more annotations—summing to a total of almost 30 thousand annotations, Fig. S1. A schematic of our pipeline for segment classification is provided in Fig. S2. Overall, the 24 topics considered for the final analysis cover about 50%-60% of cable news airtime and 20%-30% of broadcast networks news airtime, Fig. S3.

Table S2. Program classification codebook

Category	Description
Hard news	Hard news programs are focused on current events. These programs typically cover political (international and domestic), economic, and social topics. Investigative journalism about major current events and coverage of live political events both fall under "Hard news." Examples include ABC World News Tonight, 60 Minutes, and Special Report on FNC.
Talk show	Talk shows are programs where the focus is on conversation between hosts or conversation with guest(s) of the day. Examples include FNC's Fox & Friends, NBC's Meet the Press, and MSNBC's Morning Joe.
Partisan/opinion news	Partisan/opinion news shows are focused on the host's commentary about current events. While they cover similar topics to hard news programs, they tend to focus on a smaller number of news stories and spend much more time on commentary and analysis. Examples include FNC's Tucker Carlson Tonight, MSNBC's The Rachel Maddow Show, and CNN's Reliable Sources.
Soft news	In "Doing Well and Doing Good," Tom Patterson describes soft news as "typically more sensational, more personality-centered, less time-bound, more practical, and more incident-based than other news." Soft news may focus on light news content (such as celebrity and entertainment stories) or on heavier content (such as true crime). It tends to blend entertainment and journalism (and was commonly referred to as "infotainment" by academics in the 2000s). Examples include ABC's Good Morning America, NBC's Dateline, and CBS's Entertainment Tonight.
Local news	Local news content is produced by local affiliates of national networks (e.g., WPVI is Philadelphia's ABC affiliate), and the news content is specific to local markets. Examples include Eyewitness News at 6 and CBS 2 News at 6:30pm.
Other	If a program does not fit well into any of these categories, you can list it under "Other" and describe what the ideal category name should be in the "Notes" column. If possible, try to be consistent across programs when suggesting new potential categories.

Text cleaning We began by detecting ads and removing them from the transcripts. In general, television advertisements are aired repeatedly over and will have the same transcript each time. In contrast, news programs are generally broadcast once and rarely twice or three times. Using these characteristics, we were able to distinguish advertisements from news stories. Using a sliding window of three months, we divided transcripts into sentences and developed a dictionary of sentence frequencies. An advertising threshold was defined, where any group of sentences occurring more frequently than this threshold was considered an advertisement. Next, we broke down the entire program into chunks where do not cross ad breaks, and then further divided these chunks into segments of no more than 150 words in length. We first split each news program into smaller sub-documents based on the existing markers in our transcript data for story change. Next, within each sub-document, we split the text into consecutive sentences of length equal to or less than 150 words (continuing to add sentences to a segment as long as the length did not exceed 150 words).

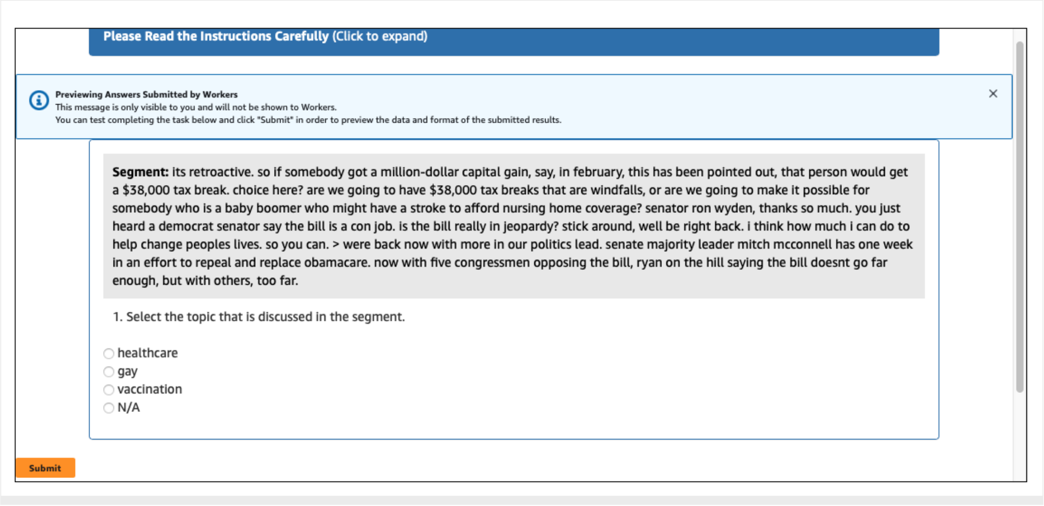

Figure S1. Annotation template.

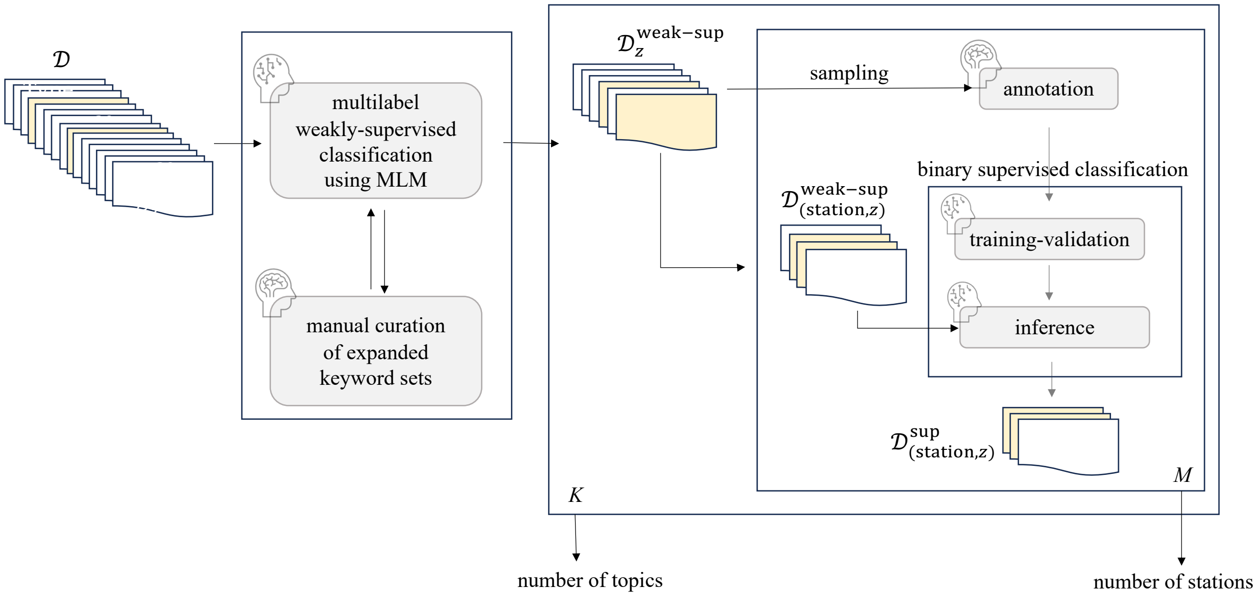

Figure S2. Topic assignment pipeline. We propose a two-layer human-in-the-loop model, where in the first layer we narrow the search space for each topic utilizing a weakly-supervised multilabel multi-class classifier applied on the whole set $\mathscr{D}$. In the second layer $\mathscr{D}_z^{sup}$ is more balanced, where for each station-topic, we use labels from human annotators as ground truth and develop supervised models.

C.1 Keyword-level multi-label category assignment

As the next step, we assigned class labels to each segment using the "class vocabulary" that had been created for each class, Table S7. One straightforward method was to identify every occurrence of each word within the "class vocabulary" for each label. This approach, however, is still subject to (i) false positives as word meanings are contextualized and not all occurrences of category keywords indicate a category, as well as (ii) false negatives due to the fact that even though the set is expanded, it is still limited and some relevant terms may not be included in the category vocabulary.

Therefore, each word in the corpus is examined within its context to determine if it fits into the corresponding category for

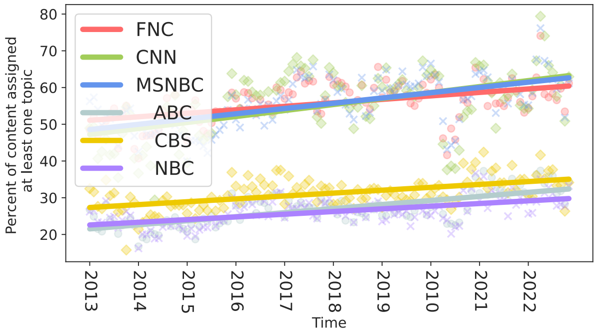

Figure S3. Proportion of news coverage that is assigned to at least one of 24 topics per station.

Algorithm S1 Expanding keyword algorithm

Input: set of all segments $\mathscr{D}$ and and one label-word w_z per topic
output: K sets of words
for z = 1:K **do**
  for i = 1:N **do**
    For each occurrence w_z in d_i make 50 predictions via a pretrained MLM
    Choose most frequent 20 words as replacements of w_z
    Manually curate the set using human experts
  end for
  Return: Set Ω_z
end for

Algorithm S2 2-layer multi-label classification

Input: Set of all segments $\mathscr{D}$ and and one set Ω_z per topic
output: multi-label vector for each segment, where a segment can belong to no topic
Parameter: Overlapping threshold γ
for z = 1:K **do**
  For each word in d_i make 50 predictions via a pretrained MLM and assign label z to d_i if more than γ% of predicted words exist in Ω_z for at least one word
  Return:$\mathscr{D}_z^{\text{weak-sup}}$
  for station $\in \{FNC, CNN, MSNBC, ABC, CBS, ABC\}$ **do**
    Annotate a random set of 50 segments from $\mathscr{D}_{(\text{station},z)}^{\text{weak-sup}}$using Amazon Mechanical Turk
    Train a supervised classifier $\mathscr{M}_z^{station}$ using annotated segments
    Using model $\mathscr{M}_z^{station}$, infer a label for each $d_i \in \mathscr{D}_{(\text{station},z)}^{\text{weak-sup}}$
  end for
  Return: $\mathscr{D}_z^{\text{sup}}$ as set of segments with topic z
end for

---

each class label. In the corpus, each label z and each document d was subjected to the following process: (1) mask each word w in document d for w that appears in the category vocabular for label z, (2) predict the top 50 words that can replace w using the same pre-trained model, (3) determine the overlap between the predicted set for word w and category vocabulary for label z, and (4) assign the class label z to document d when the overlap exceeds a threshold for at least one word in the document—the threshold can be adjusted to control precision. It should be noted that a document could have more than one label.

Model evaluation After annotating a random sample of segments, we evaluated the performance of this layer using weight-adjusted precision: percent of positive inferred labels that are actually positive. As expected, precision is higher with a tight threshold and lower with a loose threshold, Fig. S4. For our analyses in the main text, we opt for a loose threshold to include more true positives in the $\mathscr{D}_z^{\text{weak-sup}}$ set.

Table S3. Count of best performing models from model selection.

document representation	classifier	number of models
MiniLM	SVM	72
TF-IDF	SVM	58
MiniLM	RF	26
TF-IDF	RF	9

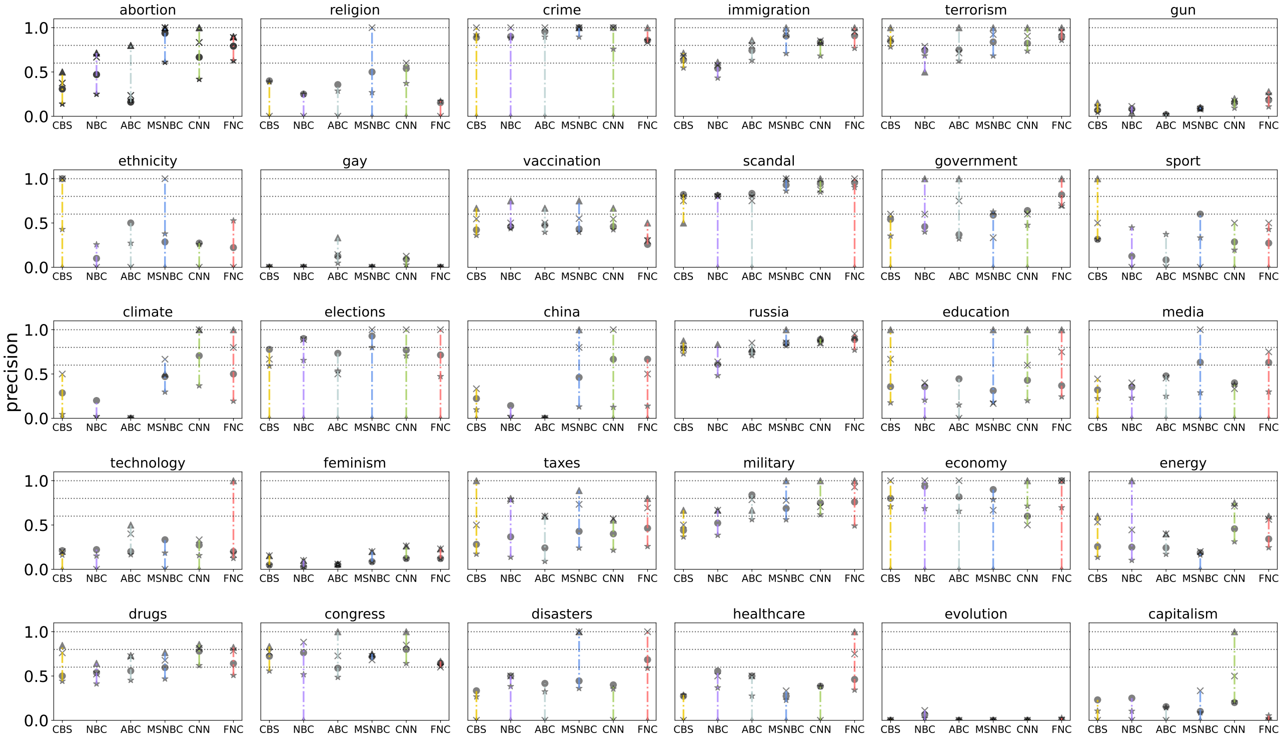

Figure S4. Weak-supervised model performance across topics and stations, with thresholds as △: 0.4, ×:0.3, ⊙:0.2

Model selection For each topic-station pair, we trained a Random Forest and an SVM model, using TF-IDF and embedding vectors from the pretrained model "all-MiniLM-L6-v2". We ran 5-fold cross-validation to train models and selected the model with the highest F1 score on the held-out set. Across all tasks, Table S3 shows the number of settings for which each document representation/model combination performed best and Fig. S5 provides the performance metrics. We dropped topics "gay," "media," "feminism," "evolution," and "capitalism" due to poor performance, as well as topic "sport" from the main text analysis.

C.2 Temporal analysis

Fig. S6 shows the proportion of airtime devoted by the six stations to each of the 24 topics, where the top row shows topics dominated by FNC (red), the second row by CNN (green) and MSNBC (blue), and the third row by broadcast news (ABC, grey; CBS, yellow; and NBC, purple). The fifth row shows topics that did not consistently receive more coverage by one source than the others. Thus, FNC consistently paid more attention to the economy, energy, immigration, government, taxes, terrorism, and religion, while CNN and MSNBC paid more attention to elections, the military, guns, abortion, ethnicity, congress, and Russia. Meanwhile, the broadcast networks paid more attention to disasters, technology, and drugs. Finally, we note that while no station paid obviously more attention to vaccination, scandals, healthcare, education, climate change, China, or crime, FNC paid distinctly less attention to climate change than any other station. In general, events impact the coverage of related topics. For example, Fig. S6 shows (1) an increase in coverage of the economy in 2017 during debate over the first Trump administration's economic policies (tariffs, tax cuts, etc.) that continued to 2019 and a spike in over the 2020–2022 period, coinciding with the COVID-19 pandemic's economic impacts; (2) surges in immigration coverage in 2014 (reflecting debate over comprehensive immigration reform proposed by Obama), 2016-2019 (in response to border policies, asylum debates, etc., during Trump's first term), and specifically on FNC in 2021 (following Biden's reversal of several Trump-era immigration

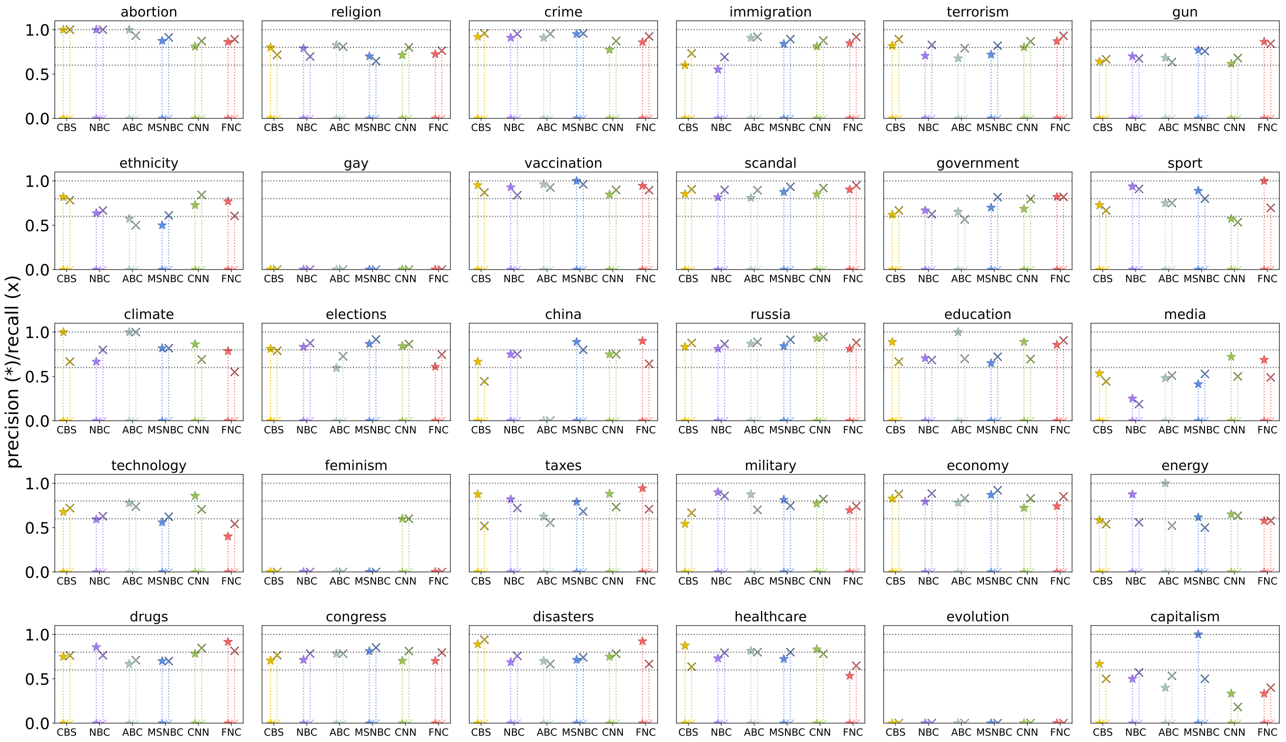

Figure S5. Model performance across topics and stations. We drop topics "gay," "feminism," "media," "evolution," and "capitalism" due to poor classification performance.

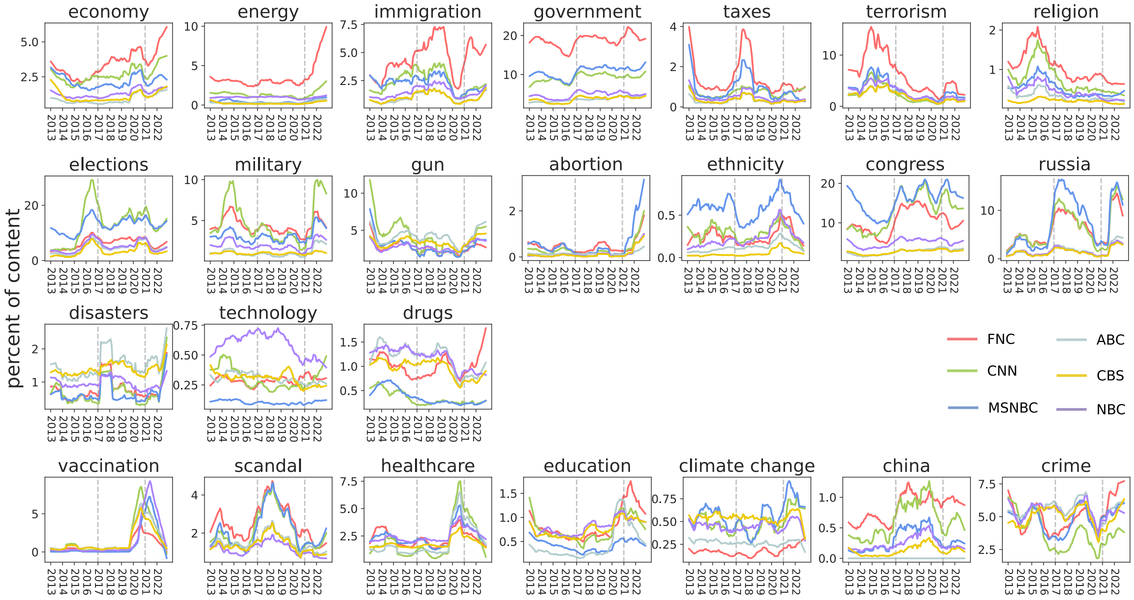

Figure S6. Topics as a percentage of channel's news content for all 24 topics.

policies); (3) an increase in coverage of vaccinations and healthcare on network news, with a spike in 2021, can be related to the pandemic; and (4) a spike in coverage of taxes in response to the 2017 Tax Cuts and Jobs Act (informally known as the Trump tax cuts), especially on FNC.

Furthermore, Fig. S7 (Fig. S7 in the SI) provides additional topic-level examples of polarization trends over time across different news stations. While there are cases where higher coverage of a topic correlates with increased polarization, this

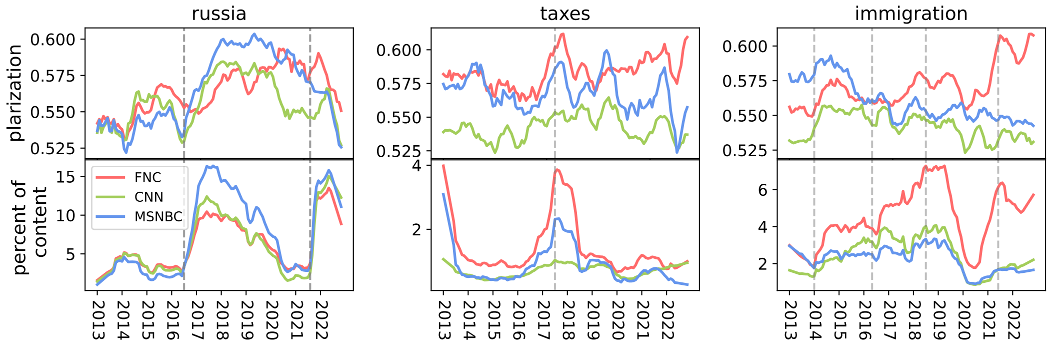

Figure S7. Temporal trends of polarization and percentage of coverage of topics Russia, taxes, and immigration.

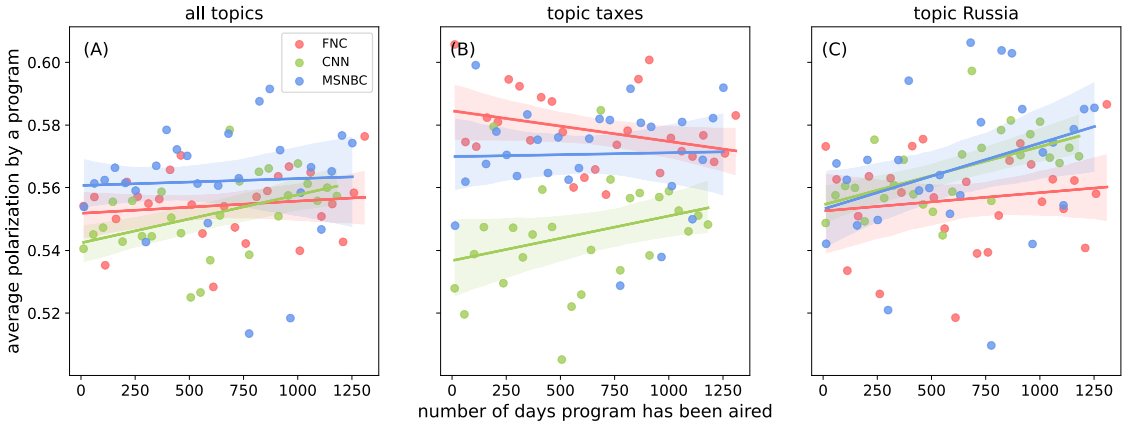

Figure S8. Polarization of news programs (y-axis) as a function of the number of days they have aired (x-axis) for: all topics (left), taxes (middle), and Russia (right).

pattern does not hold for all events. For example, in the case of the Russia topic, MSNBC exhibits the highest average polarization of the three cable stations and it remains elevated throughout Trump's first term, even as this topic occupies a shrinking share of coverage. Coverage of Russia became more polarized on FNC and CNN at the end of 2021 and early 2022, coinciding with Russia's invasion of Ukraine, but it became less polarized on MSNBC. For taxes, as well, there is a notable increase in polarization around 2017, corresponding to the Tax Cuts and Jobs Act. FNC shows the highest polarization on this topic, maintaining elevated levels afterward. Regarding immigration, coverage and polarization shift in response to major policy changes. Key events include the 2014 debate over comprehensive immigration reform proposed by Obama, the 2016–2019 period marked by Trump's border policies and asylum debates, and policy reversals under Biden in 2021. While it increases in response to each of these major events, immigration coverage on FNC reaches its highest polarization level in 2022 during Biden's presidency. Meanwhile, discussion of immigration on CNN and MSNBC shows an increase in polarization peaking in 2014, followed by a decline.

Supplementary to polarization analysis in the main text, Fig. S8 provides new analysis to understand systematically whether short-lived programs are contributing higher values of polarization, or long-lived ones (maybe be higher over, or grow over time in polarization.

C.3 Examples of partisan scores

TV news programs engage in partisan coverage filtering when they emphasize narratives and facts in their coverage of a news topic that align with the networks' and their core audiences' political predispositions[7]. We offer insight into partisan coverage filtering in practice by identifying words or phrases that are distinctive to how a particular network covers a topic. Table S4 provides more examples of segments ranging from low to high partisan score across cable stations.

Sensitivity analysis To evaluate the sensitivity of our findings to decisions about models and parameters, we replicate the results of maintext with different models and parameters. Fig. S9 and S10 show the proportion of airtime devoted by the six stations to each of the 24 topics and differences in topic selection respectively, over the outcome of the first layer. Similarly, Fig.

Table S4. Examples of high- and low-polarization segments. A high polarization score indicates that the segment can easily be assigned to the correct station by a model based on semantic content. Low polarization scores indicate that a segment is harder to assign to a station because it is less semantically distinctive. The number in parentheses after each segment is the polarization score, where 0.50 indicates that the segment could have been assigned to either station.

Topic	Polarization	Network	Excerpt from segment
Vaccination	0.46	FNC	at least ten states are lifting restrictions on certain businesses today. this afternoon at the cdc issued a report warning social distancing requirements are relaxed
	0.47	MSNBC	a we have new reporting on the cdcs decisions to relax guidelines as cases hit record numbers. plus, well have the renewed questions of what constitutes being fully vaccinated.
	0.67	FNC	the chinese communist party has been waging an information war against the united states, they have their ambassadors all around the world telling the countries that host them that this virus originated with american soldiers, not with the city of wuhan and hubei province in china.
	0.71	MSNBC	it would be hard if a politician actually sat down and made the disgustingly immoral calculations, how am i going to win this small set of anti-vaccine people. never mind it causes an outbreak.
Taxes	0.43	FNC	sources say it includes one trillion in spending cuts and one trillion in revenue also the chance that the debt ceiling would be increased one year and still yet to be determined if tax rates will go up for some americans. the president is offering a tax rate hike on those makes more than $400,000 a year.
	0.52	MSNBC	lets look at the charts. lets start with – connecticut is the focus but lets look at the whole country and a study by the tax foundation of what were the 10 highest tax states, what were the 10 lowest tax states.
	0.62	FNC	the level of contempt and disrespect the irs shows toward the tax payer leaves only one conclusion. it is time to repeal the 16th amendment, rid ourselves of the current system of taxation, put in the fair tax and by so doing eliminate not just the irs but specifically lets eliminate the lois lerner types who have forgotten who the boss really is.
	.61	MSNBC	so, poor folks need their taxes done, need tax prep, like a lot of other people, particularly earned income tax credit, whats the problem with this business? based upon predatory behavior. the problem is that its like a lot of other industries, the tax preparation industry now profits greatly from the poor.

S11 replicates Fig. 3 in the main text, over the outcome of the first layer.

Comparing partisanship of cable stations with each single broadcast station (Fig. S12) and also comparing partisanship of cable stations with all broadcast stations not conditioned on topics (Fig. S13) both show similar results as Fig. 3 in the main text.

D TV Consumption

The results of the main text are highly consistent with previous claims that cable news stations have contributed to fragmentation in the news ecosystem. Not only have cable networks as a whole diverged from their broadcast peers, both in terms of their choice of topics and the language they use to talk about them, but the cable networks have also diverged themselves along partisan lines, with FNC on the right and the increasingly similar MSNBC and CNN on the left. At the same time, however, our results also show that the "big three" broadcast networks have remained strikingly consistent, both with respect to each other and over time. ABC, NBC, and CBS, that is, tend to cover similar topics and do so using relatively similar language, and these similarities have changed little over the past decade. Thus, to the extent that viewers remain wedded to the broadcast networks shared reality may be preserved in spite of the polarization of the cable networks. Conversely, to the extent that viewers have migrated from broadcast to cable, then indeed shared reality has been diminished.

We address this concern by computing over time consumption trends for active news consumers, defined as US adults who consume at least 30 minutes of television news per month, as a fraction of the total US adult television audience (i.e. the total number of adults represented by our panel). We normalize by the adult television audience rather than the US adult population

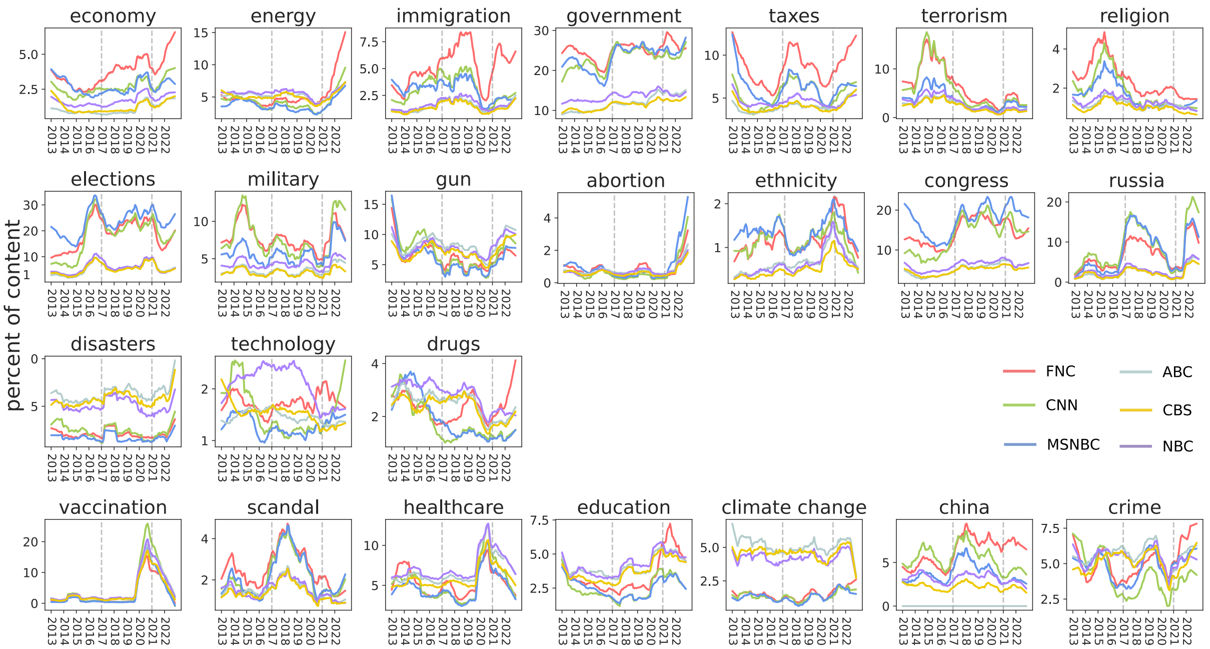

Figure S9. Topics as a percentage of station's news content for all 24 topics on the outcome of the first layer.

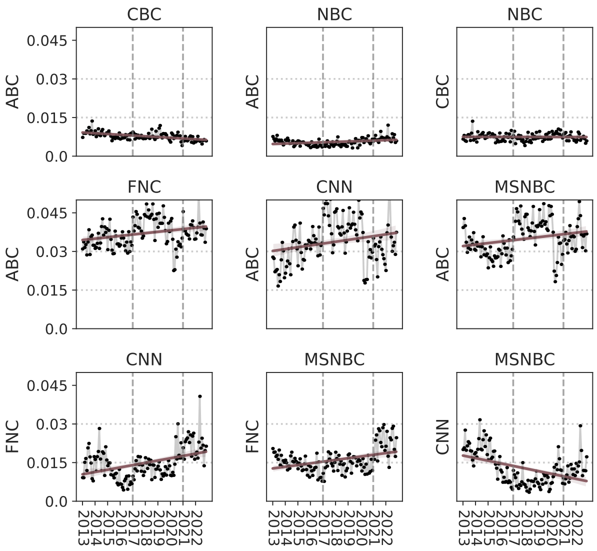

Figure S10. Average differences in coverage (fraction of time) for the 24 topics between all six stations on the outcome of the first layer.

in order to avoid confounding by so called "cord-cutting" in which households switch from cable or satellite providers to some combination of antennae and internet-based streaming services, in which case they would no longer appear in the panel. Next, we separate the overall television news audience into six "single network majority" groups, defined as viewers who allocate at least 50% of their news consumption from a single network, and a single "diverse viewers" group defined as active news consumers who do not consume a majority of their news from any one network. Table S5 shows how viewers in each of these seven groups allocate their attention across our six focal networks as well as to "other" news networks such as PBS, Spanish language networks, etc. Table S5 reveals four main insights about television news consumption. First, majority viewers for all six networks exhibit heavy concentration of consumption on their network of choice. FNC is the clear outlier with 88% concentration, where all other networks vary between 79% (for CNN) and 81% (for ABC and NBC). Second, CNN and MSNBC exhibit higher cross-consumption than either does with broadcast, and both show higher cross-consumption with

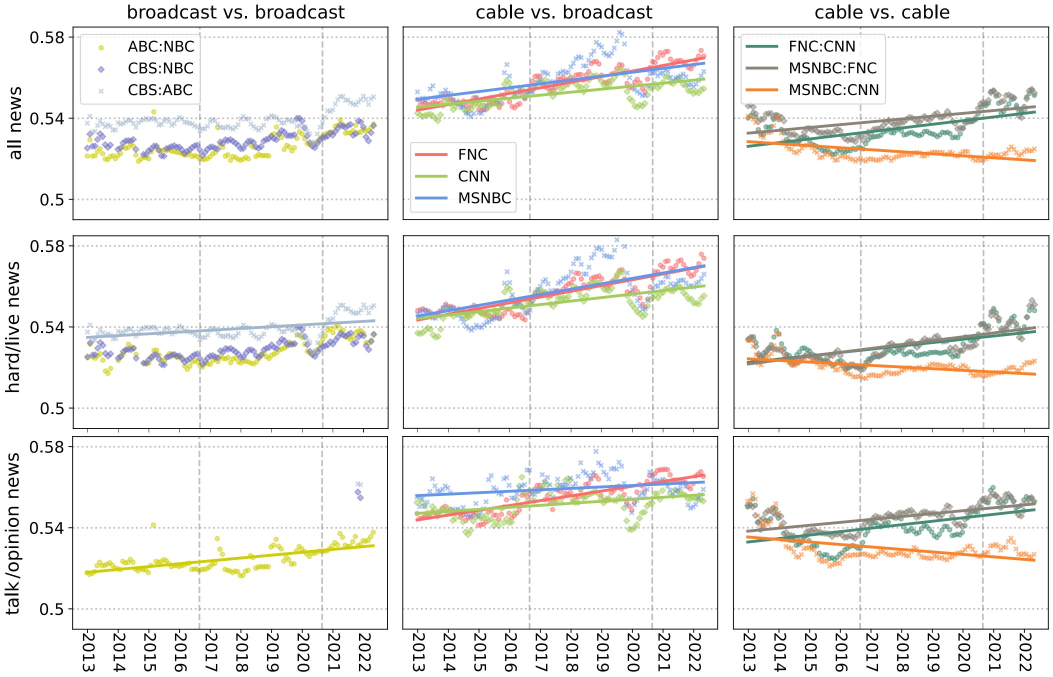

Figure S11. Polarization over time across stations on the outcome of the first layer.

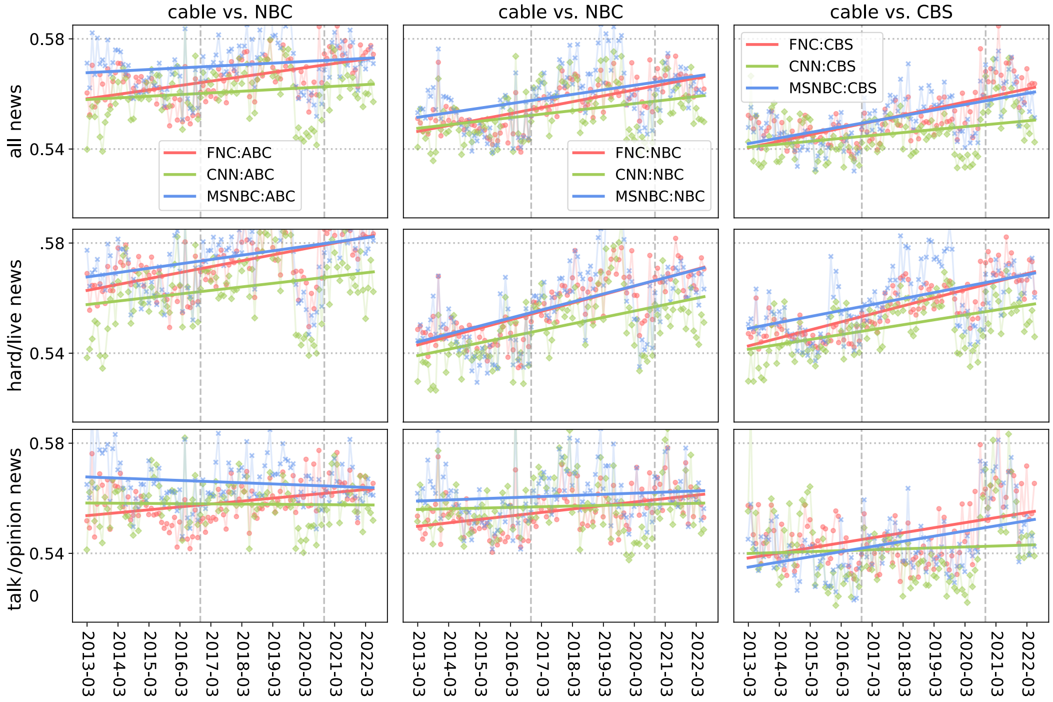

Figure S12. Polarization over time between cable and each single broadcast network.

broadcast than with FNC. Third, CBS, ABC, and NBC all exhibit higher cross-consumption among each other than any do with any cable station. Finally, diverse viewers devote the majority of their attention (61%) to the combination of broadcast stations, more than twice as much attention as they allocate to the cable news category (29%).

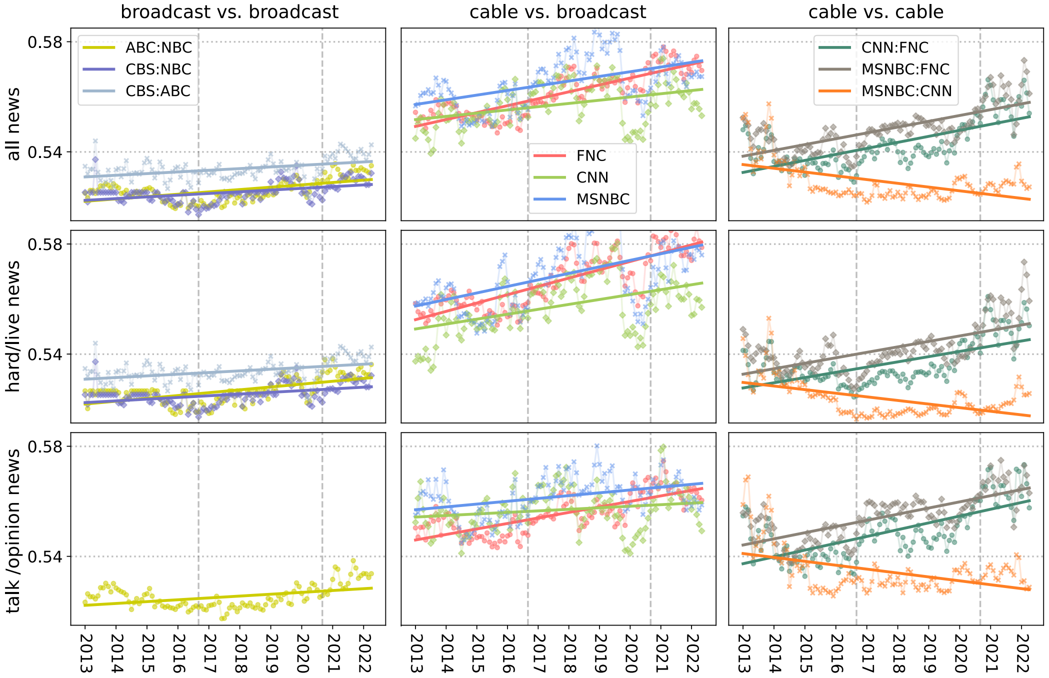

Figure S13. Polarization over time across stations, when segments are not conditioned on topic.

viewer	FNC	CNN	MSNBC	CBS	ABS	NBC	other
FNC	0.88	0.02	0.01	0.03	0.02	0.03	0.02
CNN	0.02	0.79	0.05	0.04	0.04	0.04	0.01
MSNBC	0.01	0.08	0.8	0.03	0.03	0.04	0.01
CBS	0.01	0.02	0.01	0.8	0.06	0.08	0.03
ABC	0.01	0.02	0.01	0.07	0.81	0.06	0.02
NBC	0.01	0.02	0.01	0.08	0.06	0.81	0.02
diverse	0.08	0.12	0.09	0.19	0.21	0.21	0.11

Table S5. Fraction of attention allocated to networks by single network majority viewers for each of the six focal networks as well as "diverse" viewers who do not receive more than 50% of their news from any one network.

Next, Fig. S14 shows the evolution over the time interval of the six single network majority groups in four different ways. Fig. S14A shows the share for each of the stations individually; Fig. S14B shows the sum of the shares of the three broadcast stations (grey) and the three cable stations (orange); Fig. S14C shows the share of the population that get the majority of their consumption from any combination of broadcast-only (grey) or cable-only (orange) stations; and Fig. S14D, shows the total share of the US population that consumes a majority of its news from any combination of the "big six" stations that we study here (green) as well as the total population of news consumers (black dashed line), defined as consuming at least 30 minutes of news from any source, where inset shows the percentage of the total that is accounted for by the big six. Overall, Fig. S14 reveals three main findings regarding trends in television news consumption.

First, Fig. S14A shows that the share of consumption for the three broadcast networks has generally trended down over the seven-year period of our data, whereas the share for the three cable networks has generally trended up. Consistent with prior work, the broadcast networks are roughly comparable to one another in popularity[3] while FNC is roughly the size of MSNBC and CNN combined[4]. Interestingly, all three cable networks peaked in popularity in 2020, dropping after the 2020 US presidential election, where the post-election drop for FNC is especially dramatic. In part, the overall drop likely reflects a general diminishment of interest in political news following what was an especially consequential election, but in part the drop for FNC likely reflects the widely reported audience backlash against the network for its early call of the election for Joe Biden. Interestingly, we see no equivalent drop for the broadcast networks, suggesting that the more partisan style of cable news is a benefit in politically turbulent times and a liability when the news is more "boring."

Second, Fig. S14B and C both confirm that the total audience for broadcast news has declined substantially over the

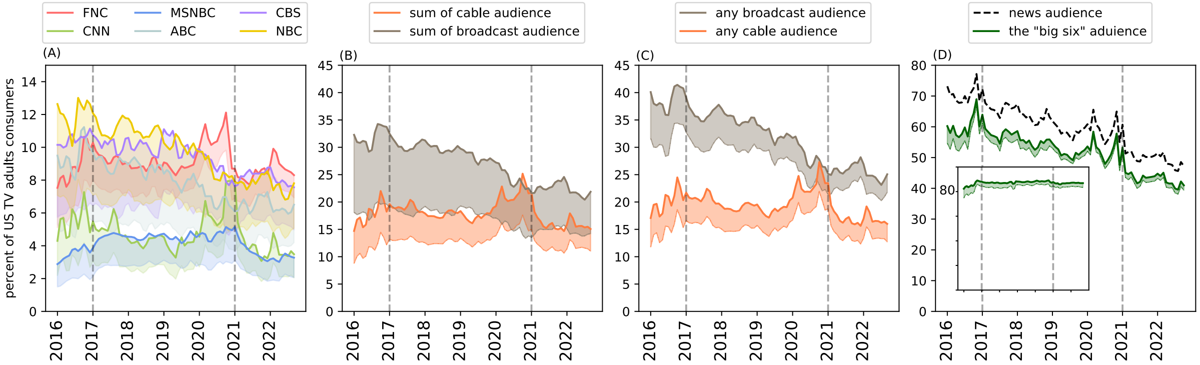

Figure S14. (A) the share of American adult TV consumers whose TV news consumption is primarily devoted to one station; (B) the sum of the shares of the three broadcast stations and the three cable stations; (C) the share of American adult TV consumers whose TV news consumption is primarily devoted to either cable (pooling FNC, MSNBC, CNN) or broadcast (pooling ABC, CBS, NBC); (D) the total share of the U.S. adult TV consumer population that consumes at least 30 minutes of news from any source and the share of Americans whose TV news consumption is primarily devoted any combination of the "big six" (inset shows the percentage of the total that is accounted for by the big six). Across all panels, solid lines represent the proportion of the U.S. adult TV consumer population that consumes more than 30 minutes of television news in a given month with 50%+ of it in that station or collection of stations, while the dotted lines represent 75%+. Vertical dashed lines reflect 2016 and 2020 presidential elections.

seven-year period while the total audience for cable has increased slightly. In both cases, however, the total broadcast audience is larger than the total cable audience for the entire period with the exception of the 2020 election, during which cable very briefly matched broadcast in popularity. After the election, the demand for cable news dropped dramatically while the decline in broadcast news has abated somewhat; thus, as of 2023 broadcast news continues to outrank cable. These trends are more pronounced in Fig. S14C, which shows the audience that gets a majority of its news from any combination of broadcast/cable stations, than in Fig. S14B which shows the simple sums of the individual audiences. The reason for this difference is that although the "diverse viewers" in Table S5, by definition, do not consume more than 50% of their news from any single network, they do allocate a majority of their attention (61%) to all broadcast networks combined; hence the population of "any broadcast" majority consumers is larger than the sum of the majority consumers for the individual networks. Because diverse viewers consume relatively little cable news, however, the population of "any cable" majority consumers is essentially just the sum of the individual networks. In other words, in spite of its decline and in contrast with conventional wisdom[5], broadcast news as a whole remains the primary source of news for Americans. This result is all the more notable in light of the far greater amount of time devoted to news by the cable networks: whereas FNN, CNN, and MSNBC all devote essentially the entire 24-hour day to news coverage, news programs only account for between 8 and 11 hours per day for ABC, NBC, and CBS (Table S1).

Third, Fig. S14D shows that, as expected, the audience getting a majority of their television news from any combination of our six stations (green) has also dropped over the seven years of our data, by roughly the same amount as the drop in the combined broadcast audience. Interestingly, however, the total audience of news consumers (dashed line) dropped even more dramatically, by roughly twenty percentage points (from 73% to 47%). Put the other way around, the fraction of the television viewing population that consumes less than 30 minutes of news per month roughly doubled, from 27% to 53%. Noting that 30 min per month is, on average, one minute per day, this result suggests a doubling in just seven years of people who do not appear to be consuming any appreciable amount of news on television. Previous work[4,6] comparing television with online news consumption has found that television dominates online by a factor of roughly five to one, and that online has not increased appreciably in recent years. Moreover, while cord cutting has eroded the "traditional" audience (i.e. cable and satellite subscribers), we note that this drop in news consumption is taking place within the population of traditional television viewers. For both reasons, this result represents a real diminishment in news consumption among the majority of Americans.

News consumption data TV consumption data is provided by Nielsen Company, which maintains large panels of American households willing to have their media habits tracked. Our analysis is based on almost six years of panel data. Individuals were limited to 24 months of participation in the panel. There are approximately 114,977 active participants in the panel on a monthly basis. The complete number of viewing events included in our data totaled more than 5.3 billion. Across all results presented in the main text and the supplementary materials, we define news consumers as adults 18 years of age or older who watch a minimum of 30 minutes of news per month. Fig. S15 provides the percentage of American adults that consume more

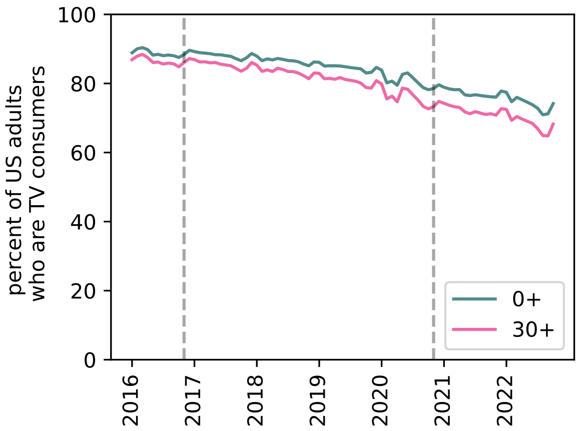

Figure S15. US adults who watch more than 30 minutes (pink) of TV a month and who watch any TV (blue). Vertical dashed lines indicate 2016 and 2020 presidential elections.

category	network
Spanish	Azteca America Affiliates, Estrella Affiliates, nivision Affiliates, nivision Deportes, Telemundo Affiliates, WAPA America, Univision Affiliates, Univision Deportes, CNN EN ESPANOL, Galavision, TUDN, UniMas, Universal Kids, Universo, NBC Universo, Telexitos, MundoMax
PBS	All PBS Stations, PBS Primary Network Affiliates, PBS Subchannels

Table S6. List of networks under Spanish and PBS category

than 30 minutes of television and the percentage that consume any television per month. Fig. S20 shows the breakdown of percent of news consumers with non-zero min news consumption per month who receive the majority (A) or most (B) of their news from one of the defined set of stations.

Sensitivity analysis We replicate the consumption analysis of Fig. S14 with a more lenient definition of news consumers, defined as adults who consume any news on TV, Fig. S16 and a more strict definition of news consumer, defined as adults who consume more than 2 hours of news per month, Fig. S18. Each of these figures news consumers normalized to adult TV consumers and the total US adult population. Fig. S14 is also replicated with the total US adult population as the denominator, Fig. S17. Additionally, Figs. S19 and S19 provide a breakdown of the share of news consumers with 30+ min news consumption per month, which receive a majority (left) or most (right) of their news from one of the defined set of stations (Spanish, PBS, and “rest” [Table S6]).

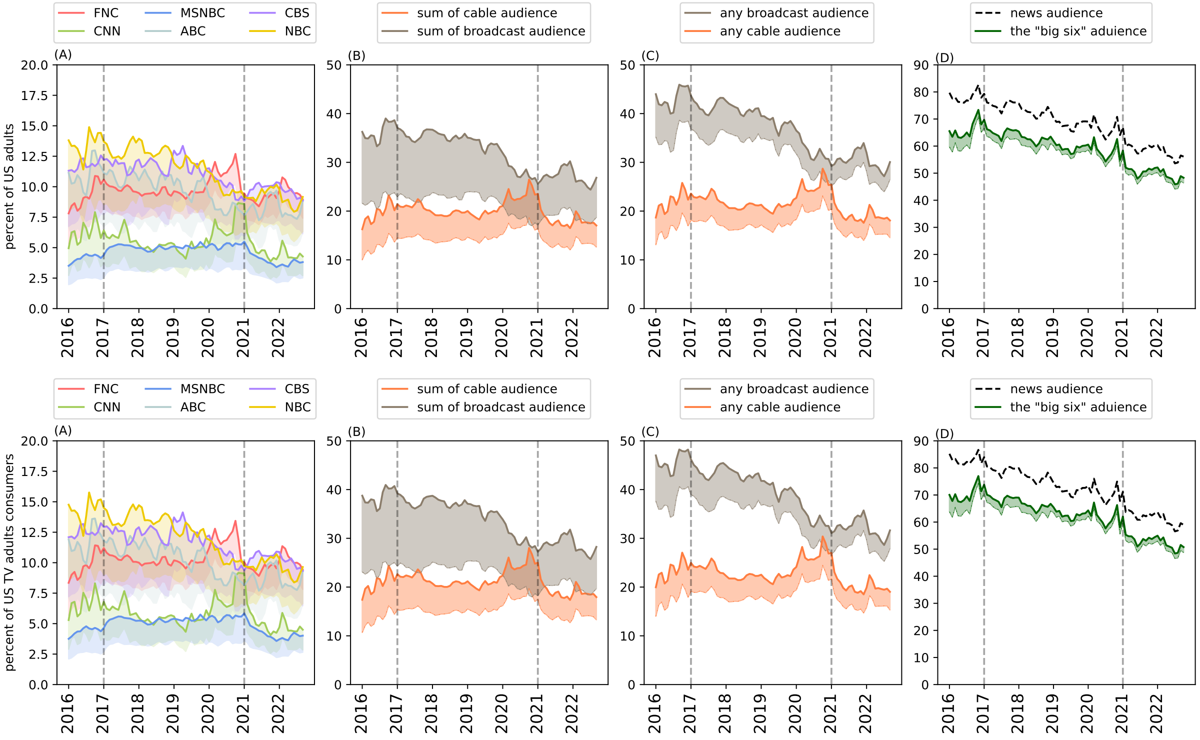

Figure S16. (A) the share of American adults (top) and American adult TV consumers (bottom) whose TV news consumption is primarily devoted to one station; (B) the sum of the shares of the three broadcast stations and the three cable stations; (C) the share of American adults (top) and American adult TV consumers (bottom) whose TV news consumption is primarily devoted to either cable (pooling FNC, MSNBC, CNN) or broadcast (pooling ABC, CBS, NBC); (D) the total share of the American adults (top) and American adult TV consumers (bottom) that consumes any TV news from any source and the share of Americans whose TV news consumption is primarily devoted any combination of the "big six" (inset shows the percentage of the total that is accounted for by the big six). Across all panels, solid lines represent the non-zero consumption of television news in a given month with 50%+ of it in that station or collection of stations, while the dotted lines represent 75%+. Vertical dashed lines reflect 2016 and 2020 presidential elections.

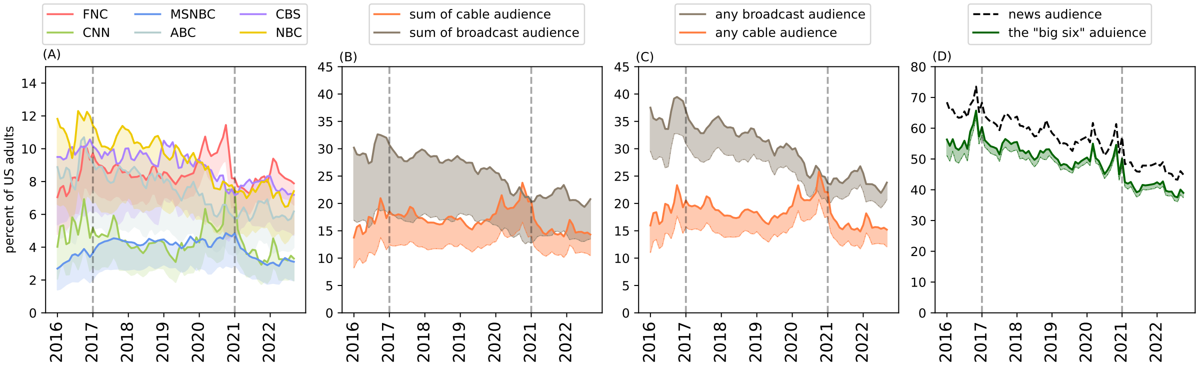

Figure S17. (A) the share of American adults whose TV news consumption is primarily devoted to one station; (B) the sum of the shares of the three broadcast stations and the three cable stations; (C) the share of American adults whose TV news consumption is primarily devoted to either cable (pooling FNC, MSNBC, CNN) or broadcast (pooling ABC, CBS, NBC); (D) the total share of the US population that consumes any news from any source and the share of Americans whose TV news consumption is primarily devoted any combination of the "big six" (inset shows the percentage of the total that is accounted for by the big six). Across all panels, solid lines represent more than 30 minutes of television news in a given month with 50%+ of it in that station or collection of stations, while the dotted lines represent 75%+. Vertical dashed lines reflect 2016 and 2020 presidential elections.

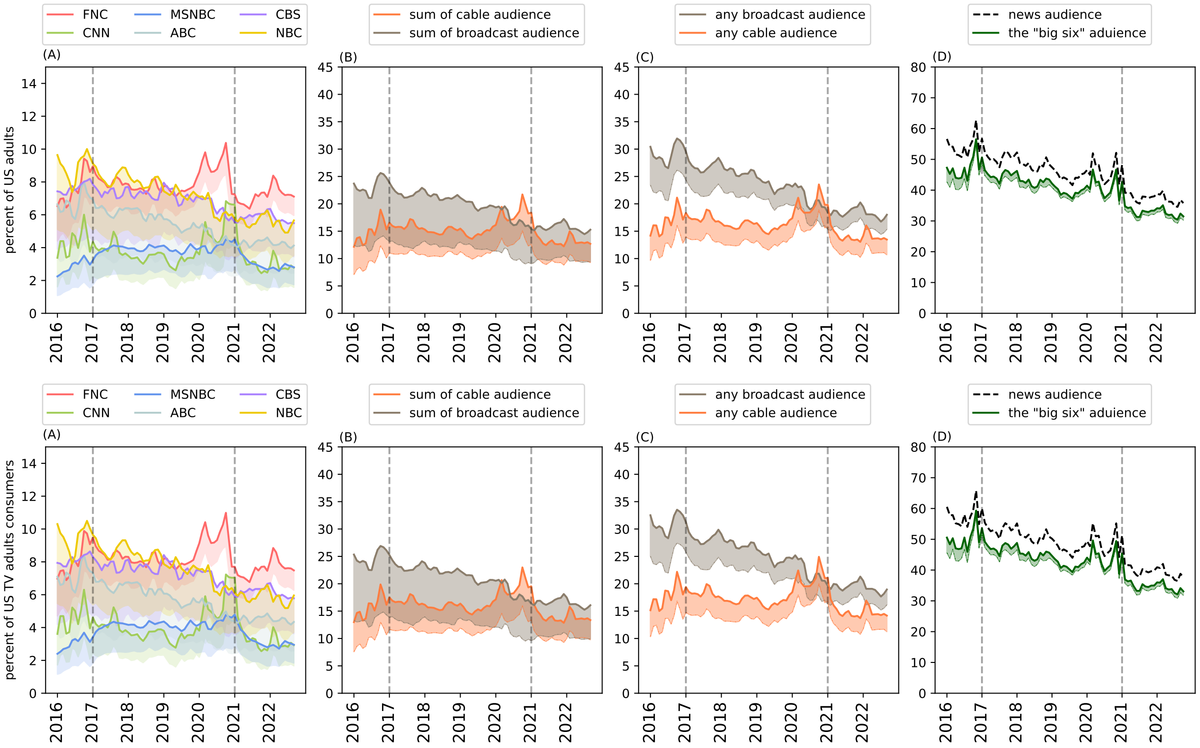

Figure S18. (A) the share of American adults (top) and American adult TV consumers (bottom) whose TV news consumption is primarily devoted to one station; (B) the sum of the shares of the three broadcast stations and the three cable stations; (C) the share of American adult (top) and American adult TV consumers (bottom) whose TV news consumption is primarily devoted to either cable (pooling FNC, MSNBC, CNN) or broadcast (pooling ABC, CBS, NBC); (D) the total share of the American adult (top) and American adult TV consumers (bottom) that consumes at least 120 minutes of news from any source and the share of Americans whose TV news consumption is primarily devoted any combination of the "big six" (inset shows the percentage of the total that is accounted for by the big six). Across all panels, solid lines represent more than 120 minutes of television news in a given month with 50%+ of it in that station or collection of stations, while the dotted lines represent 75%+. Vertical dashed lines reflect 2016 and 2020 presidential elections.

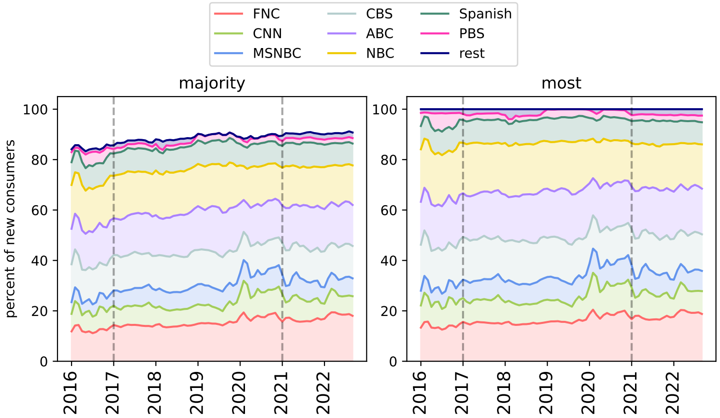

Figure S19. Breakdown of the share of news consumers with 30+ min news consumption per month, which receive a majority (A) or most (B) of their news from one of the defined set of stations.

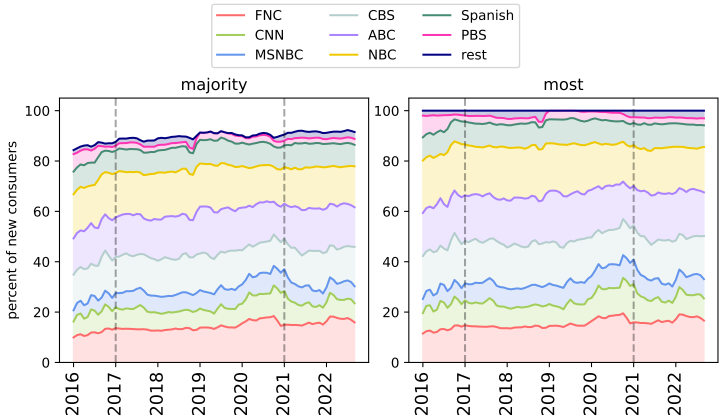

Figure S20. Breakdown of percent of news consumer with non-zero min news consumption per month which receive majority (A) or most (B) of their news from one of the defined set of stations.

Table S7. Label and extended vocabulary lists.

Label	Extended vocabulary list
abortion	abortion, pregnant, orgasm, conception, fetal, womb, childbirth, ultrasound, adultery, reproduction, termination, unnatural, choice, newborn, erection, divorce, condom, reproductive, liberty, maternity, conceived
religion	religion, religious, faith, belief, religions, chapel, beliefs, worship, prayer, temple, cult, christianity, denomination, believers, conversion, doctrine, scripture, believer, confession, ritual, catholic, mosque, protestant, holy, spirituality, islamic, muslims, hinduism, spiritual, catholicism, churches, philosophy
crime	crime, crimes, mob, offense, offence, criminals, killer, sin, victim, murderer, committed, crack, offenders, disorder, sentence, murders, offender, guilty, conviction, stolen, commit, murdered
immigration	immigration, immigrants, migration, aliens, deportation, emigration, asylum, alien, visa, integration, border, annexation, deported, indigenous, passport, illegally, enclave, internment, influx
terrorism	terrorism, terrorist, terrorists, qaeda, attacks, kidnapping, militants, bombing, aggression, jihad, militant, isis, isil, bombs, iraq, guerrilla, bombings, hamas, threat, conflict, bomber, israel, raid, trafficking, insurgents
gun	gun, pistol, shot, handgun, firearm, firearms, rifle, bullet, revolver, sword, knife, barrel, pistols, shotgun, kill, child, cannon, gunshot, trigger, shoot, bullets, rifles, ammunition
ethnicity	ethnicity, descent, heritage, ancestry, location, origin, racial, identify, origins, colour, colored, tribe, han, territory, background, accent, height, hispanic, minorities, lineage, nationalities, surname, linguistic, identities, demographics, dialect, african
gay	gay, queer, homosexual, homo, homosexuality, sexual, heterosexual, married, wed, dating, bisexual, openly, relationship, couple, ho
vaccine	vaccine, disease, infection, vaccines, flu, cure, testing, mortality, influenza, outbreak, cdc, immunity, prevalence, casualty, patient, screening, illness, virus, malaria, deaths, epidemic, fever, smallpox, viral, dose,covid
scandal	scandal, scandals, controversy, allegations, investigation, corruption, expose, leak, gossip, leaks, controversies, revelations, rumor, leaking, drama, embarrassment, irregularities, settlement, secret, embarrassing, disgrace, revelation, fallout, misconduct, bribery, alleged, dirt, myth, abuse, disclosure, mess
government	government, governmental, govt, ministry, politician, agency, regime, department, governor, governing, officials, dod, authority, federal, rule, funding, coalition, grant
sport	sport, sporting, club, competition, championship, play, contest, track, pursuit, hobby, cup, ball, tournament, football, match, skate, catch, sportsman, pool, athlete, dance, soccer, player, racing, hockey, craft, league, basketball, coach, arena, gym, olympic, olympics, freestyle, stadium, professional, baseball

climate	climate, weather, warming, cold, climatic, global, spring, freeze, cooling, atmosphere, freezing, greenhouse, climates, cool, temperature, ice, millennium, geo, eco, snow, environmental, rain, storm, water, sustainable, cap, atmospheric, rising, sustainability, catastrophe, extreme, earth
elections	elections, election, votes, candidates, voters, polls, results, primaries, parties, elected, campaigns, reelected, electors, democrats, winners, debates, offices, republicans, reelection, elect, primary, presidential, ballots, republican, outcomes, tickets, democratic, terms, administrations, majority, referendum, campaign, inauguration, runs, unopposed, sweeps, vote, voted, victories, candidate, presidents, assemblies, districts, voter
china	china, beijing, asia, canton, shanghai, india, province, hua, thailand, prc, shandong, bei, tibet, wu, mao, korea, sino, taiwan, jiang, malaysia, xinjiang
russia	russia, russian, moscow, russians, putin, ukraine, soviet, rus, vladimir, ussr, germany, nato, sergei, crimea, ru, dmitry, syria
education	education, educational, educated, schooling, educate, instruction, elementary, educators, learning, college, educating, academy, educator, children, academic, curriculum, grade, studies, students, classrooms, scholarship, graduation, course, instructional, enrollment, teaching, teachers, tuition
media	media, press, tv, radio, newspaper, television, newspapers, network, journalist, publications, networks, reporters, papers, paper, communications, publicity, youtube, headlines, twitter, journalists, site, social, sky, facebook, com, online, bbc, website, reporter, broadcasting, herald, sites, websites, propaganda, headline, sources, google, broadcaster, publishing, popular, journalism, polling, stories, financial, multimedia, mobile, ap
technology	technology, technologies, tech, software, technological, hardware, devices, computer, gear, chip, invention, method, systems, facility, knowing, technical, platform, tool, machines, mechanism, computing, smart, electronic, electronics, capability, feature, plant, procedure, concept, functionality, intel, tools, theory, standard, wireless, privacy, capabilities, machinery, algorithm
feminism	feminism, feminist, woman, porn, icon, left, sorority, liberation, breast, chick, revolution, wnba, feminine, domination, girl, goddess
taxes	taxes, taxation, taxpayers, rates, subsidies, fines, laws, customs, tariffs, taxpayer, utilities, duties, benefits
military	military, soldiers, soldier, special, naval, strong, troop, defensive, militia, general, heavy, reserve, tank, infantry, garrison, strategic, marines, trained, unit, tactical, cavalry, artillery, base, personnel, fort, missile, battalion, fighting, corps, northern, helicopter, combat
economy	economy, economies, economics, savings, currency, empire, dollar, bubble, gdp, market, agriculture, prosperity, trade, fast, production, recession, consumer, economist, markets, industries, businesses, economical, workforce, industrial, crop, boom, exchange, sector, capitalism, rich, rate, equilibrium, enterprise, account, banks, unemployment, cash, economically, agenda

energy	energy, energies, fuel, electric, resource, coal, electrical, efficiency, petroleum, thermal, hydro, ethanol
drugs	drugs, narcotics, substances, heroin, cocaine, pills, chemicals, substance, medications, weed, alcohol, cigarettes, cannabis, addiction, drugged, drinks, dealers, liquor, drink, possession, needles, med, ecstasy, bottles
congress	congress, legislators, chamber, congressman, representatives, committees, convention, assembly, congresses, representative, legislatures, senators, chambers, senator, delegation, volunteers, session, chair, select, parliamentary, california, commons, hall, representation, passage, executive, directors, floor, judiciary
disasters	disasters, hurricanes, floods, catastrophic, accidents, crises, tsunami, earthquakes, tornadoes, devastating, fires, storms, situations, disastrous, hurricane, emergencies, explosions, cyclones, flooding, incidents, waves, hazards, lois, earthquake, burns, hail, victims, tornado, damage, collapses, trauma, flood, injuries, crashes, failures, casualties, devastation, devastated
healthcare	healthcare, hospitals, surgery, medicare, healing, hospice, pension, physician, surgical, doctors, physicians, nhs, nursing, surgeon, dental, ems, retirement, doctor
evolution	evolution, evolve, evolving, progression, evolutionary, evolved, transformation, emergence, shift, adaptation, division, creation,beginning, genesis, mutation, shifting, phenomenon, innovation, extension, revival, biology, existence, darwin, extinction
capitalism	capitalism, capitalist, socialism, imperialism, greed, communism, globalization, marxist, marxism, corporate, regulation, socialist, liberal, poor, liberalism, boycott, greedy, marx, inequality, utopia, entrepreneurship, communist, ci, mo, fascism, finance, bourgeois, civilization, slavery

References

1. Yin, W., Hay, J. & Roth, D. Benchmarking zero-shot text classification: Datasets, evaluation and entailment approach. *CoRR* **abs/1909.00161** (2019). 1909.00161.
2. Hugging Face. Nli-based zero shot text classification (2022).
3. Pew Research Center. Network news fact sheet. Tech. Rep., Washington, D.C. (2023).
4. Muise, D. *et al.* Quantifying partisan news diets in web and tv audiences. *Sci. Adv.* **8**, eabn0083 (2022).
5. Yglesias, M. The case for fox news studies. *Polit. Commun.* **35**, 681–683, DOI: 10.1080/10584609.2018.1477532 (2018).
6. Allen, J., Howland, B., Mobius, M., Rothschild, D. & Watts, D. J. Evaluating the fake news problem at the scale of the information ecosystem. *Sci. advances* **6**, eaay3539 (2020).
7. Broockman, D. & Kalla, J. The impacts of selective partisan media exposure: A field experiment with fox news viewers. *OSF Prepr.* **1** (2022).
